# Characterization of a broadly specific cadaverine *N*-hydroxylase involved in desferrioxamine B biosynthesis in *Streptomyces sviceus*

**DOI:** 10.1371/journal.pone.0248385

**Published:** 2021-03-30

**Authors:** Lesley-Ann Giddings, George T. Lountos, Kang Woo Kim, Matthew Brockley, Danielle Needle, Scott Cherry, Joseph E. Tropea, David S. Waugh

**Affiliations:** 1 Department of Chemistry, Smith College, Northampton, MA, United States of America; 2 Department of Chemistry & Biochemistry, Middlebury College, Middlebury, VT, United States of America; 3 Basic Science Program, Frederick National Laboratory for Cancer Research, Frederick, MD, United States of America; 4 Center for Structural Biology, Center for Cancer Research, National Cancer Institute, Frederick, MD, United States of America; Weizmann Institute of Science, ISRAEL

## Abstract

*N*-hydroxylating flavin-dependent monooxygenases (FMOs) are involved in the biosynthesis of hydroxamate siderophores, playing a key role in microbial virulence. Herein, we report the first structural and kinetic characterization of a novel alkyl diamine *N*-hydroxylase DesB from *Streptomyces sviceus* (*Ss*DesB). This enzyme catalyzes the first committed step in the biosynthesis of desferrioxamine B, a clinical drug used to treat iron overload disorders. X-ray crystal structures of the *Ss*DesB holoenzyme with FAD and the ternary complex with bound NADP^+^ were solved at 2.86 Å and 2.37 Å resolution, respectively, providing a structural view of the active site environment. *Ss*DesB crystallized as a tetramer and the structure of the individual protomers closely resembles the structures of homologous *N*-hydroxylating FMOs from *Erwinia amylovora* (DfoA), *Pseudomonas aeruginosa* (PvdA), and *Aspergillus fumigatus* (SidA). Using NADPH oxidation, oxygen consumption, and product formation assays, kinetic parameters were determined for various substrates with *Ss*DesB. *Ss*DesB exhibited typical saturation kinetics with substrate inhibition at high concentrations of NAD(P)H as well as cadaverine. The apparent *k*_*cat*_ values for NADPH in steady-state NADPH oxidation and oxygen consumption assays were 0.28 ± 0.01 s^-1^ and 0.24 ± 0.01 s^-1^, respectively. However, in product formation assays used to measure the rate of *N*-hydroxylation, the apparent *k*_*cat*_ for NADPH (0.034 ± 0.008 s^-1^) was almost 10-fold lower under saturating FAD and cadaverine concentrations, reflecting an uncoupled reaction, and the apparent NADPH K_M_ was 33 ± 24 μM. Under saturating FAD and NADPH concentrations, the apparent *k*_*cat*_ and K_M_ for cadaverine in Csaky assays were 0.048 ± 0.004 s^-1^ and 19 ± 9 μM, respectively. *Ss*DesB also *N*-hydroxylated putrescine, spermidine, and L-lysine substrates but not alkyl (di)amines that were branched or had fewer than four methylene units in an alkyl chain. These data demonstrate that *Ss*DesB has wider substrate scope compared to other well-studied ornithine and lysine *N*-hydroxylases, making it an amenable biocatalyst for the production of desferrioxamine B, derivatives, and other *N*-substituted products.

## Introduction

*N*-hydroxylating monooxygenases (NMOs) play an important role in the biosynthesis of iron-chelators or siderophores. Microorganisms express these flavin-dependent enzymes under iron-limiting conditions to produce siderophores to sequester iron, an essential nutrient required for cell growth and development. Using the electron donor NADPH and molecular oxygen, NMOs catalyze the first committed step in hydroxamate siderophore biosynthesis by oxidizing a primary amine in alkyl amines [1, 2] and amino acids, such as lysine [3] and ornithine [4, 5], to *N*-hydroxylated products. Subsequent acylation of these products form hydroxamates, imparting metal-chelating properties. NMOs are involved in the biosynthesis of a variety of hydroxamate siderophores in many microorganisms, such as *Aspergillus fumigatus* (SidA) [4], *Pseudomonas aeruginosa* (PvdA) [6, 7], *Bordetella bronchiseptica* RB5 (AlcA) [8], and *Streptomyces pilosus* (DesB) [9]. Deleting genes encoding these enzymes in pathogens, such as *A*. *fumigatus* [10] and *P*. *aeruginosa* [11], inhibit siderophore production as well as their growth in low iron media. Thus, characterizing NMOs can provide insights into combating pathogen virulence and identifying potential drug targets for the development of antimicrobial agents.

**Fig 1:**
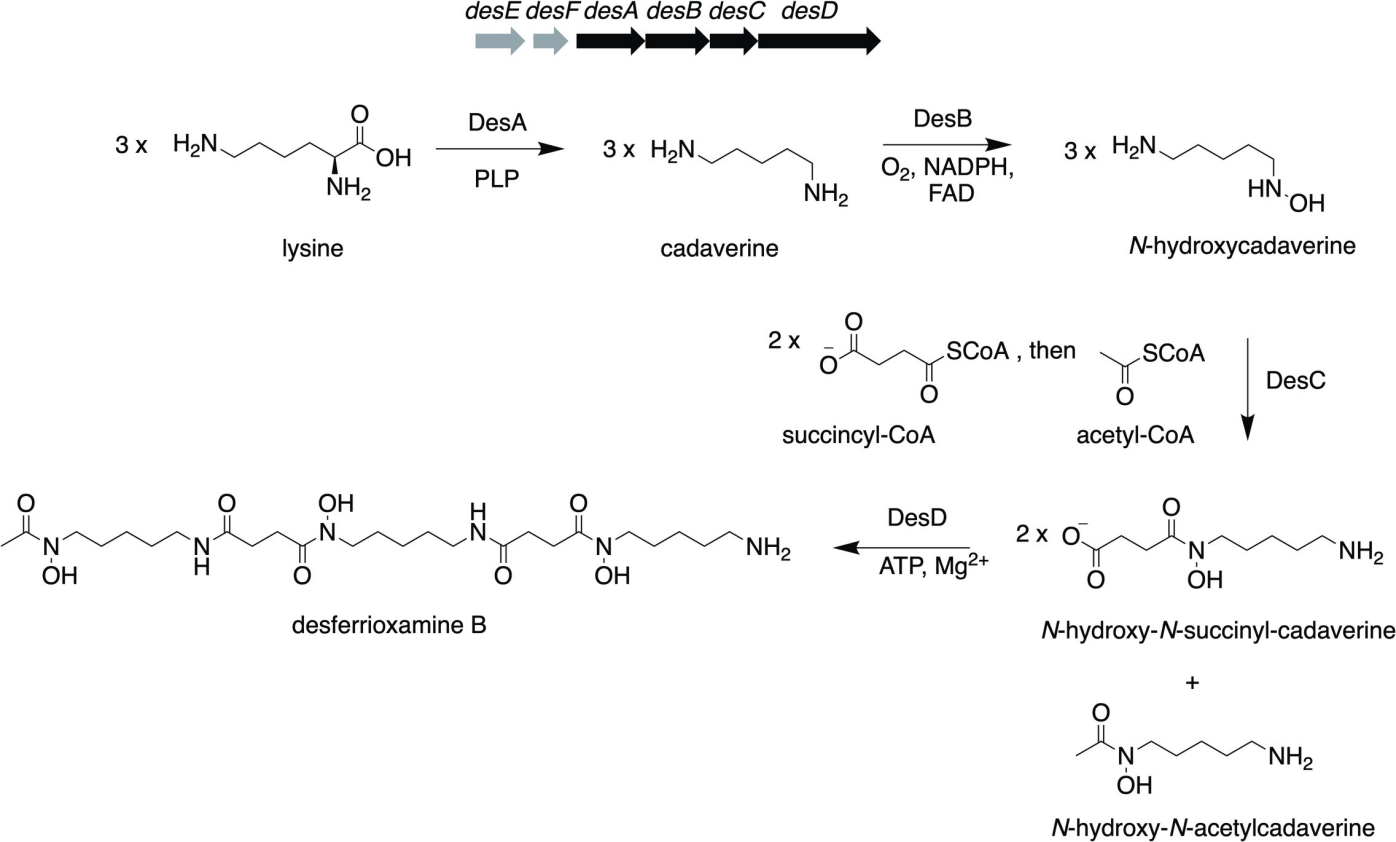
Desferrioxamine B biosynthetic pathway in *S*. *sviceus*. Organization of the biosynthetic gene cluster in *S*. *sviceus* and proposed enzymatic steps involved in the synthesis of desferrioxamine B. Genes encoding the four desferrioxamine biosynthetic enzymes are in black (*desA*, *desB*, *desC*, and *desD*) and genes involved in ferrioxamine uptake and utilization are in grey (*desE* and *desF*).

In addition to serving as drug targets against pathogenic microbes, *N*-hydroxylases are indicative of the biosynthesis of hydroxamate siderophores, some of which are used in clinical treatments [12, 13]. The lysine-derived desferrioxamine B (Fig 1) is a tris-hydroxamate siderophore produced by aerobic, Gram-positive soil actinomycetes, such as *S*. *coelicolor* M145 [12] and *S*. *pilosus* [14], clinically used for the intravenous treatment of iron poisoning and hemochromatosis [15, 16]. The hydroxamate moiety is the key pharmacophore [17] and also the reason why desferrioxamine B is widely used as a hexadentate chelator in immunoPET and cell tracking [13]. Desferrioxamines are biosynthesized by an operon encoding a lysine decarboxylase (DesA), cadaverine *N*-hydroxylase (DesB), acyl transferase (DesC), and an ATP-dependent nonribosomal peptide synthetase (DesD) [18] (Fig 1). A ferric siderophore lipoprotein receptor (DesE) and a ferric-siderophore hydrolase (DesF) are also found within the gene cluster in various strains of *Streptomyces* (Fig 1) [19, 20]. These genes were first identified in *S*. *coelicolor* [18] and have since been found in most sequenced *Streptomyce*s genomes [20], several species of *Salinispora* [21], as well as plant pathogen *E*. *amylovora* [2]. While a number of publications have provided insight into the activities of DesA [22], DesB [2], DesC [23], and DesD [23, 24] from *S*. *pilosus* and *S*. *coelicolor*, DesA is the only enzyme to be kinetically characterized [22]. Homologs of these biosynthetic enzymes have been identified in several clinical pathogens. For example, there are a number of DesB homologs (36–60% sequence identity) in clinical pathogens, such as *Streptococcus pneumonia*, *Acinetobacteria baumannii*, and *Yersinia pestis* (S1 Fig in S1 File). Thus, understanding the mechanism of siderophore biosynthetic enzymes, such as DesB, can lead to the identification of new bioactive hydroxamate siderophores and improve the production of other hydroxamates and molecules containing N-O functional groups [25].

We aimed to determine the structure and characterize the kinetics of DesB from *S*. *sviceus* (*Ss*DesB) to understand the limitations of desferrioxamine biosynthesis and evaluate the substrate scope of alkyl diamine *N*-hydroxylases. Alkyl diamine *N*-hydroxylases, such as the putrescine *N*-hydroxylases GorA from *Gordonia rubripertincta* CWB2 [1] (38% sequence similarity to *Ss*DesB) and PubA from *Shewanella oneidensis* MR-1 [26] (51% sequence similarity to *Ss*DesB) (S2, S3 Figs in S1 File), have a wider substrate scope compared to well-known ornithine and lysine NMOs, which are specific for their substrates and NADPH. For example, GorA is active with NAD(P)H, FAD, and either putrescine, cadaverine, or 1,6-diaminohexane substrates [1]. PubA also *N*-hydroxylates either putrescine and cadaverine in the presence of FAD and NADPH [26]. The *N*-hydroxylation step catalyzed by both GorA and PubA are catalytically similar to that of *Ss*DesB but remain to be kinetically characterized with both cadaverine and putrescine substrates.

Although a crystal structure of the *Ss*DesB homolog DfoA with bound FAD and NADP^+^ (53% sequence identify) from the plant pathogen, *E*. *amylovora* [2], was recently reported at 2.8 Å resolution, visualization of the electron density maps deposited in the Protein Data Bank (PDB; PDB code: 5O8R) revealed a poor fit of the FAD and NADP^+^ cofactors into the experimental electron density. A more accurate view of the enzyme active site with bound cofactors is needed. Furthermore, the activity and cofactor specificity of DfoA was solely evaluated by NADPH oxidation assays [2]. There was no analysis of the *N*-hydroxylation step, which is critical for understanding product formation. Herein, we heterologously expressed and purified *Ss*DesB and used X-ray crystallography as well as NAD(P)H oxidation, O_2_ consumption, and hydroxamate product formation assays to kinetically and structurally characterize this NMO, which has a higher sequence identity to DesB in the industrial desferrioxamine B-producing *S*. *pilosus* strain. Steady-state kinetics were performed with putrescine and cadaverine to understand the substrate specificity of *Ss*DesB. We hypothesized that *Ss*DesB would have a broader substrate scope similar to GorA and a comparable three-dimensional structure to other well-studied ornithine (PvdA from *P*. *aeruginosa*, 31% sequence identity) and alkyl diamine *N*-hydroxylases (DfoA).

## Materials and methods

### General materials

An *E*. *coli* codon-optimized, *N*-terminal hexahistidine-tagged *SSEG_08523* gene encoding *Ss*DesB was synthesized and subcloned into pET28a (+) by GenScript Corp. (Piscataway, NJ). *N*-hydroxycadaverine was purchased from Enamine Ltd. (Kyiv, Ukraine). All other materials were purchased from Sigma-Aldrich (St. Louis, MO) or Thermofisher Scientific (Waltham, MA) and used without further purification.

### General methods

Microplate kinetic assays were monitored on an Epoch BioTek microplate spectrophotometer (Winooski, VT). The *Ss*DesB extinction coefficient was determined using an Agilent 8453 diode array UV-vis spectrophotometer (Santa Clara, CA). High resolution electrospray ionization mass spectrometry (ESI-MS) was performed on an Acquity Ultra Performance Liquid Chromatography (UPLC) I-Class System in tandem with a Waters Xevo quadrupole-time of flight (q-TOF) mass spectrometer. Oxygen consumption assays were monitored on an Oxygraph Plus system (Hansatech; Norfolk, UK). DNA sequencing was performed by Eurofins Genomics (Louisville, KY).

### Protein expression of *Ss*DesB with *N*-terminal hexahistidine tag

The *Ss*DesB gene cloned into pET28a (+) was transformed into *Escherichia coli* BL21 Star (DE3) One ShotⓇ cells (Invitrogen; Carlsbad, CA) according to the manufacturer’s instructions. Cells were plated on Luria-Bertani (LB)-agar containing 50 μg/mL kanamycin and grown overnight at 37°C. A single colony was used to inoculate LB broth (100 mL) containing 50 μg/mL kanamycin, which was grown overnight at 37°C at 200 rpm. The overnight culture was used to inoculate 1-L of LB broth containing 50 μg/mL kanamycin, which was grown at 30°C at 200 rpm. Cells were induced with 1 mM isopropyl β-D-1 thiogalactopyranoside (IPTG) once an optical density at 600 nm (OD_600_) of 0.6 was reached and grown for four additional hours before being harvested at 4,500 rpm and stored at -80°C.

### Protein purification of *Ss*DesB with *N*-terminal hexahistidine tag

Cells were lysed twice via the French Press at 20,000 psi after incubation with lysozyme (1 mg/ml) and protease inhibitor cocktail (P8849; Sigma-Aldrich, St. Louis, MO) 50 mM phosphate buffer (pH 8) containing 300 mM sodium chloride and 10 mM imidazole. After centrifugation at 9,100 rpm for 1 hr at 4°C, the supernatant was filtered with a 0.22 μM Millex-GS syringe filter (EMD Millipore; Burlington, MA) and incubated with HisPur^TM^ Ni-NTA resin (Thermofisher; Waltham, MA). The resin was equilibrated with 50 mM phosphate buffer (pH 8) containing 300 mM sodium chloride and 20 mM imidazole. Protein was eluted in 50 mM phosphate buffer (pH 8) containing 300 mM sodium chloride, 125 mM imidazole, and 2.5% glycerol. Using a 10 K Amicon Ultra centrifugal filter unit (Thermofisher Scientific; Waltham, MA), protein was buffer-exchanged into 100 mM phosphate buffer containing 1 mM dithiothreitol and 10% glycerol and stored at -80°C.

### Determination of *Ss*DesB extinction coefficient

*Ss*DesB (156 μM) was heated in triplicate at 95°C for 15 min and clarified by centrifugation at 13,000 rpm for 10 min at room temperature. After the supernatant was removed and reheated again, its absorbance was measured over a range of 200–800 nm. Using Beer’s Law (A = εcl) and the extinction coefficient of FAD (11,300 M^-1^cm^-1^), the extinction coefficient of DesB was determined, as the concentration of the enzyme should be equivalent to the concentration of free FAD after denaturation. Furthermore, the percent of FAD bound was also determined using Eq (1).

$$\%of\;FAD–bound\;enzyme=\frac{\left[Free\;FAD\right]}{\left[Enzyme\right]}\times 100\%$$ (1)

### NAD(P)H oxidation assays

Steady-state kinetic parameters for NAD(P)H in oxidation assays were determined by incubating *Ss*DesB (0.7 μM) with 50 μM FAD, and 10 mM cadaverine at 25°C. Assays were initiated after 5 min with varying concentrations of NAD(P)H (5.5 μM–1 mM), resulting in a total volume of 250 μL. The decrease in absorbance at 340 nm was monitored using an Epoch BioTek microplate spectrophotometer. The initial rate at which the enzyme oxidized NAD(P)H over time was determined using Beer’s Law (*ε*_NAD(P)H_ = 6,220 M^-1^cm^-1^ at 340 nm) and an NAD(P)H standard curve (to determine the quality of cofactor and pathlength of the microplate).

### Oxygen consumption assays

Molecular oxygen consumption consumed by *Ss*DesB was monitored using a Hansatech Oxygraph (Norfolk, England, UK). Steady-state kinetic parameters for substrates were obtained by incubating substrate of various concentrations, 100 mM phosphate buffer, pH 8, *Ss*DesB (7.4 μM), and 50 μM FAD for 5 min at 25°C with stirring. Reactions were then initiated by the addition of 0.7 mM NADPH (700 μL total volume). Steady-state kinetic parameters for NADPH were obtained by incubating 100 mM phosphate buffer, pH 8, *Ss*DesB (7.4 μM), 50 μM FAD, and 0.7 mM cadaverine for 5 min incubation with stirring at 25°C. Assays were initiated with various NADPH concentrations (9.1 μM–9.3 mM).

### Detection of hydrogen peroxide

Standard assays (400 μL total volume) consisting of 100 mM phosphate buffer pH 8, 50 μM FAD, 2.12 μM *Ss*DesB, 10 mM cadaverine, and 0.7 mM NADPH were incubated at 25°C. Assays with boiled enzyme as well as no enzyme were also prepared. After 30 s and 72 s, aliquots were removed from assays and the amount of hydrogen peroxide produced was quantified via the QuantiChrom^TM^ peroxide assay kit (BioAssay Systems; Hayward, CA) according to the manufacturer’s instructions. A hydrogen peroxide standard curve was used to quantify the amount of hydrogen peroxide produced by *Ss*DesB.

### Cloning and protein expression of *Ss*DesB for structural studies

The *SsDesB* gene was amplified from *E*. *coli* codon-optimized DNA obtained from GenScript Corp. (Piscataway, NJ) (*SSEG_08523*, pET28a (+)) by polymerase chain reaction (PCR) using the following oligonucleotide primers: 5’-GGC TCG GAG AAC CTG TAC TTC CAG ACC GCG CGT CCG GAG -3’(PE-3112) and 5’-GGG GAC CAC TTT GTA CAA GAA AGC TGG GTT ATT ACA CGC TAA ACT CCT GGA ACG C-3’ (PE-3113). The PCR amplicon was then used as the template for a second PCR amplification with primer PE-277 (5’-GGG GAC AAG TTT GTA CAA AAA AGC AGG CTC GGA GAA CCT GTA CTT CCA G -3’) and PE-3113. The amplicon from the second PCR, coding for *Ss*DesB (T2-V425) with an *N*-terminal tobacco etch virus (TEV) recognition site (ENLYFQ/T), was recombined into the entry vector pDONR 221 (Life Technologies; Grand Island, NY) using Gateway cloning technology to generate the entry clone pGL3067, and the nucleotide sequence was confirmed by Sanger sequencing [27]. The modified *SsDesB* gene was then recombined from pGL3067 into pDEST527 to generate the expression vector pGL3070. This plasmid produces an *N*-terminal hexahistidine-tagged *Ss*DesB that can be cleaved by TEV protease to yield the *Ss*DesB enzyme [25]. The protein was expressed in the *E*. *coli* strain Rosetta2(DE3). Cultures were grown to mid-log phase (OD_600_ ~ 0.5) at 37°C in Luria Bertani broth containing 0.2% glucose, 100 μg ml^-1^ ampicillin, and 30 μg ml^-1^ chloramphenicol. Overexpression of the *Ss*DesB fusion protein was induced with 1 mM isopropyl β-D-1-thiogalactopyranoside for 18–20 h at 18°C. Cells were pelleted by centrifugation and stored at -80°C.

### Protein purification for structural studies

All purification steps were performed at 4–8°C. *E*. *coli* cell paste (10 g) was resuspended in 200 mL of ice-cold lysis buffer containing 50 mM Tris-HCl pH 7.4, 200 mM NaCl, 25 mM imidazole, and cOmplete, EDTA-free protease-inhibitor cocktail tablets (Roche Diagnostics; Mannheim, Germany). Cells were lysed by passing through an APV-1000 homogenizer (Invensys APV Products, Albertslund, Denmark) at 69 MPa three times, and the lysate was centrifuged for 30 min at 15,500 rpm. The supernatant was filtered by vacuum through a 0.2 μm polyethersulfone membrane and applied to a 5-mL HisTrap FF column (GE Healthcare, Piscataway, NJ) pre-equilibrated with lysis buffer. The column was washed to baseline with lysis buffer and protein was eluted using a linear gradient from 25 mM to 500 mM imidazole. Fractions containing the His_6_-*Ss*DesB fusion protein were combined and concentrated using an Ultracel 30 kDa ultrafiltration disc (EMD Millipore, Billerica, MA). The concentrated protein was diluted with 50 mM Tris pH 7.4, 200 mM NaCl buffer to achieve an imidazole concentration of 25 mM. The His_6_-*Ss*DesB fusion protein was then digested with 5 mg polyhistidine-tagged TEV protease overnight [27]. The digest was applied to a second HisTrap column (2 × 5 mL) pre-equilibrated with lysis buffer. The deep-yellow colored fractions containing *Ss*DesB were combined and half was concentrated to 12–15 mg/mL in the lysis buffer mentioned above. Aliquots were flash frozen in liquid nitrogen and stored at -80°C for use in structural studies. The protein was judged to be >90% pure by sodium dodecyl sulfate-polyacrylamide gel electrophoresis (SDS-PAGE), and the molecular weight was confirmed by electrospray ionization mass spectrometry. The remaining portion of protein was incubated overnight at 4°C with 10 mM DTT. The sample was concentrated, as above, and fractionated on a HiPrep 26/60 Sephacryl S-300 HR column (GE Healthcare; Piscataway, NJ) equilibrated with a buffer containing 25 mM Tris-HCl pH 7.4, 150 mM NaCl, and 2 mM tris(2-carboxyethyl)phosphine (TCEP). The peak fractions of recombinant *Ss*DesB were combined and concentrated to 12–15 mg/mL. Aliquots were flash frozen in liquid nitrogen and stored at -80°C for use in biochemical studies. This protein was judged to be >95% pure by SDS-PAGE using a Coomassie stain and its molecular weight was confirmed by electrospray ionization mass spectrometry.

### Product formation assays

Using *Ss*DesB without an *N*-terminal hexahistidine tag, the amount of *N*-hydroxylated product formed in *Ss*DesB assays at 25°C was determined using a modified Csaky iodine oxidation assay [4, 28]. In a 96-well plate, *Ss*DesB (1 μM) was incubated in 100 mM phosphate buffer, pH 8.0 with 50 μM FAD and varied amounts of substrate (cadaverine or putrescine) for 5 min before reactions were initiated with 0.7 mM NAD(P)H (400 μL total volume). For assays in which NAD(P)H concentrations were varied (0.031–4 mM), the substrate concentration was 10 mM. Hydroxylamine standards (9.4–300 μM) in 100 mM phosphate buffer, pH 8.0, were also prepared. Aliquots of enzyme assays (61.5 μL) as well as standards were removed and quenched with perchloric acid (0.03 N). Samples were then centrifuged at 7,000 rpm for 3 min at room temperature before the supernatant (47 μL) was removed and added to equal volumes of 10% w/v sodium acetate (47 μL) and 0.6% w/v sulfanilic acid in 25% acetic acid (47 μL). Samples were then incubated with 0.07% w/v iodine in glacial acetic acid for 15 min with shaking before the addition of 19 μL of sodium thiosulfate (0.02 N) and 19 μL of 1-napthylamine (0.6% w/v). After 45 min of shaking at 25°C, 96-well plates were read at 562 nm using an Epoch Biotek microplate spectrophotometer.

### Substrate scope assays

Standard assays (400 μL total volume) consisting of 100 mM phosphate buffer pH 8, 50 μM FAD, 1 μM *Ss*DesB (without an *N*-terminal hexahistidine tag), 10 mM substrate, and 0.7 mM NADPH were incubated at 25°C. The following substrates were assayed: cadaverine, putrescine, L-lysine, 1,3-diaminopropane, spermidine, L-ornithine, *n*-butylamine, and 3-dimethylaminopropylamine.

### Liquid chromatography/mass spectrometry (LC/MS) analysis

Standard assays (400 μL) consisting of 100 mM phosphate buffer pH 8, 10 mM substrate, *Ss*DesB (1.0 μM, without an *N*-terminal hexahistidine tag) and 0.7 mM NADPH were incubated at 25°C. Assays without substrate were also prepared as a negative control. Aliquots (50 μL) were removed after 10 min, quenched with twice the volume of HPLC-grade acetonitrile (Thermofisher; Waltham, MA), and chilled at -20°C for 10 min. After centrifugation at 12,500 rpm for 3 min at room temperature, the supernatant was incubated with 50 μL of 100 mM borate buffer pH 8 followed by the addition of 20 μL of 10 mM Fmoc-Cl dissolved in LC/MS grade methanol. After 5 min, samples were incubated with 20 μL of 0.1 M 1-adamantylamine in 1:1 acetonitrile: water for an additional 10 min to remove excess Fmoc-Cl. Samples were then analyzed by LC/MS. Using a flow rate of 0.6 mL/min, samples (1 μL) were injected onto an Acquity UPLC BEH C18 column (2.1 × 50 mm × 1.7 μm) (Waters; Milford, MA) with an Acquity precolumn filter (2.1 mm × 0.2 μm) attached. The column was warmed to 40°C and UPLC separation was achieved using a 5–100% gradient of acetonitrile: water with 0.1% formic acid over 9 min. ESI-MS in sensitivity mode was used to obtain exact mass measurements of Fmoc-derivatized substrates and products. The following ESI settings were used: 3 kV capillary voltage, 45 V sample cone voltage, 120°C source temperature, 550°C desolvation temperature, 50 L/hr cone gas flow, and 800 L/hr desolvation gas flow. A leucine enkephalin reference (Waters; Milford, MA) was used for the lock spray with a 30 s reference scan frequency, 45 V reference cone voltage, 6 eV collision energy, and a 99.9 dynamic range enhancement setting.

### Kinetic data analysis

Kinetic data were analyzed in Kaleidagraph (Synergy; Reading, PA) and Prism 8 (GraphPad; San Diego, CA). The *k*_cat_ and K_M_ of *Ss*DesB with specific substrates were determined by fitting initial rate data to the Michaelis-Menten Eq (2). The substrate inhibition constant (*K*_i_) was determined for cadaverine and NAD(P)H (with the exception of oxygen consumption initial rate data with varied NADPH) by fitting the initial rate data to the Haldane Eq (3). All reaction rates were measured such that no more than 10% product formed in enzyme assays. See the S1 File for fitted data.

$$v=\frac{k_{cat}[S]}{K_{M}+[S]}$$ (2)

$$v=\frac{k_{cat}[S]}{K_{M}+[S]+\frac{{\left[S\right]}^{2}}{K_{i}}}$$ (3)

### Crystallization

Purified *Ss*DesB (12 mg/mL in 50 mM Tris-HCl, pH 7.4, 200 mM NaCl, and 25 mM imidazole) was screened for crystals using several sparse-matrix crystallization screens from Hampton Research, Microlytic, Qiagen, and Molecular Dimensions using a Gryphon crystallization robot (Art Robbins Instruments; Sunnyvale, CA). Optimization screens of the initial crystallization screening hits were carried out using the hanging-drop vapor diffusion method in Easy-Xtal 15-well plates (Qiagen; Germantown, MD). Additional optimization screens were completed using the Hampton Research Additive screen. Crystals of *Ss*DesB for data collection were obtained by mixing 2.5 μL of protein solution (12.1 mg/mL) with 2 μl well solution (0.1 M Hepes pH 7.5, 0.2 M sodium chloride, 25% w/v polyethylene glycol 3350), and 0.5 μL of 0.1 M taurine and sealing over 500 μL of well solution. The trays were incubated at 4°C. Yellow, plate-like crystals appeared within 1 week. A single crystal was retrieved from a drop using a Litholoop (Molecular Dimensions; Maumee, OH) and transferred to a 1 μl drop of Paratone-N where excess mother liquor was whisked away from the crystal. The crystal was removed with a Litholoop and flash-cooled by plunging it into liquid N_2_.

Crystals of *Ss*DesB in complex with NADP^+^ were obtained by incubating 12.1 mg/mL of protein with 1 mM NADP^+^ (Sigma-Aldrich, St. Louis, MO) for 2 hrs at 4°C. Precipitated material was removed by centrifugation for 10 min and 2.5 μL of the *Ss*DesB:NADP^+^ mixture was mixed with 2.0 μL of well solution (0.1 M Hepes pH 7.5, 0.2 M sodium chloride, 25% w/v polyethylene glycol 3350) and 0.5 μL (0.1 M sarcosine) and sealed over 500 μL of well solution. After 1 hr, the drops were streak-seeded with a whisker using previous crystals of *Ss*DesB as a seed source. Yellow, plate-like crystals appeared within 2 days. A single crystal for data collection was retrieved using a Litholoop, transferred to a 1 μL drop of Paratone-N, where excess mother-liquor was whisked away from the crystal, and the crystal was flash-cooled by plunging into liquid N_2_.

### Data collection

X-ray diffraction data for crystals of *Ss*DesB and *Ss*DesB complexed with NADP^+^ were collected on the 22-ID and 22-BM beamlines, respectively, of the SER-CAT facilities at the Advanced Photon Source, Argonne National Laboratory, Argonne, Illinois. For the *Ss*DesB crystal, 360 images were collected using a wavelength of 1.0000 Å, an oscillation angle of 1.0°, a crystal to detector distance of 300 mm, and an exposure time of 0.25 s. Data were collected from a single crystal of *Ss*DesB complexed with NADP^+^ using a wavelength of 1.0000 Å, a crystal-to-detector distance of 300 mm, an oscillation angle of 1.0°, and an exposure time of 20 s. A total of 180 frames of data were collected. All X-ray diffraction images were processed with *HKL-3000* [29].

The 2.37 Å structure of *Ss*DesB in complex with NADP^+^ was solved by molecular replacement with the program *PHASER* [30] in the *PHENIX* [31] suite and using the structure of *E*. *amylovora* DfoA [2] (PDB entry 5o8r, chain A, 53% sequence identity) as a search model after removing all solvent and ligand molecules. Based on the Matthews coefficient of 2.36 Å^3^ Da^-1^ and solvent content of 47.9%, a search for 8 molecules in the asymmetric unit was performed [32–34]. Iterative rounds of model-rebuilding were performed manually using *Coot* [35] followed by additional automated model adjustment using the PDBredo server [36]. The PDB coordinate files for the FAD and NADP^+^ ligands were prepared using *Molinspiration* (https://molinspiration.com), and the ligand restraint files used during refinements were prepared with the program *eLBOW* [37] in *PHENIX*. Refinements were performed with *phenix*.*refine* [38]. Water molecules were automatically located with *Coot*, manually inspected, and refined with *phenix*.*refine*.

The structure of *Ss*DesB at 2.86 Å resolution was solved by molecular replacement with *Phaser* using chain A of the *Ss*DesB-NADP^+^ complex structure after removing all solvent and ligand molecules and searching for 8 molecules in the asymmetric unit (2.30 Å^3^ Da^-1^ and solvent content of 46.5%). Iterative rounds of manual model rebuilding were performed using *Coot* followed by refinement with *phenix*.*refine*. All structures were validated using the validation tools available in *Coot* and also with the *Molprobity* server [39]. Refinement statistics and model validation are outlined in Table 4. The coordinates and structure factor files were deposited in the Protein Data Bank under accession codes 6XBB and 6XBC.

## Results

### Protein purification and characterization of FAD cofactor

The purification yielded 30–40 mg of a 51.7 kDa *N*-terminal hexahistidine tagged protein from 1 L of culture (S4A Fig in S1 File). The UV-visible absorbance spectrum of *Ss*DesB indicated the presence of the FAD cofactor bound to the purified enzyme with characteristic absorbance maxima at 370 nm and 457 nm (S4B Fig in S1 File). The extinction coefficient at 457 nm was 14,478 M^-1^cm^-1^ and ~15% of flavin was estimated to copurify with *Ss*DesB.

### NAD(P)H oxidation and coenzyme specificity

Kinetic parameters of *Ss*DesB NAD(P)H oxidation activity was determined by monitoring the decrease in the absorbance of NAD(P)H at 340 nm (Table 1). At a constant concentration of cadaverine (10 mM), *Ss*DesB oxidized NADPH with an apparent *k*_cat_ of 0.28 ± 0.01 s^-1^ and *k*_cat_/K_M_ of 43 ± 11 mM^-1^s^-1^. NADH was oxidized with an apparent *k*_cat_ of 0.38 ± 0.09 s^-1^ and *k*_cat_/K_M_ of 3.3 ± 1.3 mM^-1^s^-1^, indicating a preference for oxidizing NADPH. There was also evidence of substrate inhibition when assayed with either cadaverine or NAD(P)H at concentrations above 0.7 mM.

**Table 1:** Steady-state kinetic parameters determined in NAD(P)H oxidation assays for different substrates.

Substrate	*k*_cat_, s^-1^	*K*_m_, μM	*k*_cat_/*K*_m_, mM^-1^ s^-1^	*K*_I_, mM
Cadaverine (0.7 mM NADH)	0.38 ± 0.07	79 ± 23	4.8 ± 1.6	2.2 ± 1.8
NADPH (10 mM Cadaverine)	0.28 ± 0.01	6.4 ± 1.6	43 ± 11	
NADH (10 mM Cadaverine)	0.38 ± 0.09	120 ± 36	3.3 ± 1.3	2.9 ± 3.4
Putrescine (0.7 mM NADH)	0.28 ± 0.02	140 ± 23	2.1 ± 0.3	

Initial rates measured with varied concentrations of cadaverine or putrescine (or 10 mM) in NAD(P)H oxidation assays in the presence of *Ss*DesB, 50 μM FAD, and 0.7 mM NAD(P)H (or varied concentrations). All measurements were made in triplicate.

### Oxygen consumption assays varying NADPH, cadaverine, and putrescine

**Fig 2:**
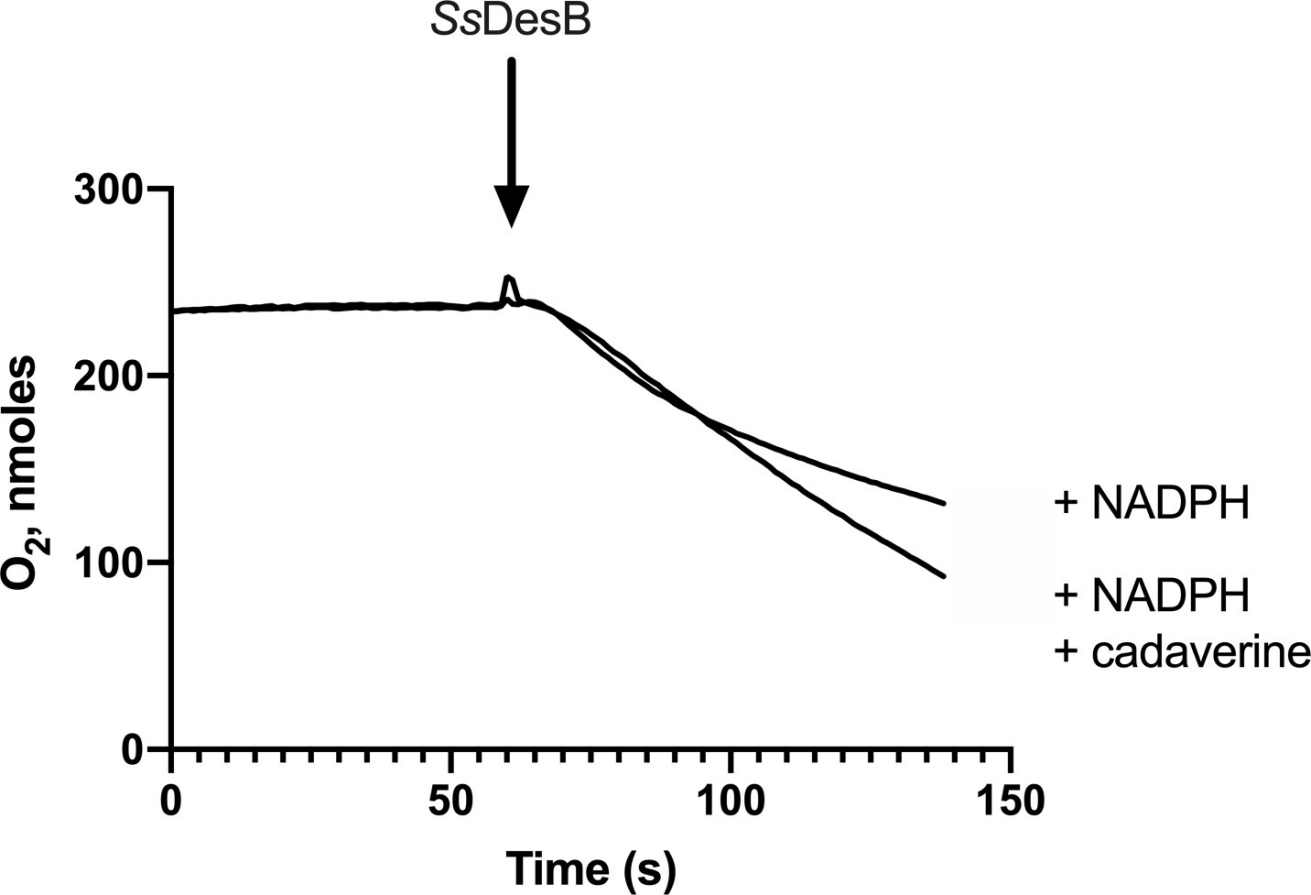
Oxygen consumption assays. Oxygraph traces demonstrate high oxidase activity in the absence of substrate. Assays contained 100 mM sodium phosphate buffer, pH 8 with 0.7 mM NADPH and 0.05 mM FAD in the absence or presence of 10 mM cadaverine.

The activity of *Ss*DesB was assessed by monitoring its rate of oxygen consumption with NAD(P)H, FAD, and various substrates. Substrate-independent oxidation was observed as *Ss*DesB consumed slightly more oxygen (1.5-fold more) with cadaverine (Fig 2). Similar kinetic parameters were determined using the oxygen consumption assay compared to those obtained from NAD(P)H oxidation assays (Table 2). At a constant cadaverine concentration of 10 mM and varying NADPH concentrations, *Ss*DesB consumed molecular oxygen with an apparent *k*_cat_ of 0.24 ± 0.01 s^-1^ and *k*_cat_/K_m_ of 2.9 ± 0.4 mM^-1^s^-1^. These values are somewhat consistent with those determined in NADPH oxidation assays. When the concentration of NADPH was held constant and cadaverine concentrations were varied, *Ss*DesB consumed molecular oxygen with an apparent *k*_cat_ of 0.20 ± 0.01 s^-1^ and *k*_cat_/K_m_ of 4.3 ± 0.1 mM^-1^s^-1^. When putrescine concentrations were varied at constant NADPH concentrations, *Ss*DesB consumed molecular oxygen with a similar apparent *k*_cat_ of 0.29 ± 0.01 s^-1^ but lower catalytic efficiency of 0.62 ± 0.15 mM^-1^s^-1^. QuantiChrome assays detected peroxide formation in only assays with enzyme (11 μM in 30 s and 20 μM in 72 s) and none in control assays without enzyme or with boiled enzyme (S5 Fig in S1 File). The presence of peroxide indicated that *Ss*DesB does not use all of its reduced molecular oxygen to form an *N*-hydroxylated product.

**Table 2:** Steady-state kinetic parameters determined in oxygen consumption assays for different substrates.

Substrate	*k*_cat_, s^-1^	*K*_m_, μM	*k*_cat_/*K*_m_, mM^-1^s^-1^	*K*_*I*_, mM
Cadaverine(0.7 mM NADPH)	0.20 ± 0.01	4.7 ± 1	4.3 ± 0.1	37 ± 4
NADPH(10 mM Cadaverine)	0.24 ± 0.01	83 ± 10	2.9 ± 0.4	
Putrescine(0.7 mM NADPH)	0.29 ± 0.02	470 ± 110	0.62 ± 0.15	

Initial rates measured with varied concentrations of cadaverine or putrescine (or 10 mM) in the presence of *Ss*DesB, 50 μM FAD, and 0.7 mM NADPH (or varied concentrations). All measurements were made in triplicate.

### *N*-hydroxylation activity varying NAD(P)H, cadaverine, and putrescine

**Fig 3:**
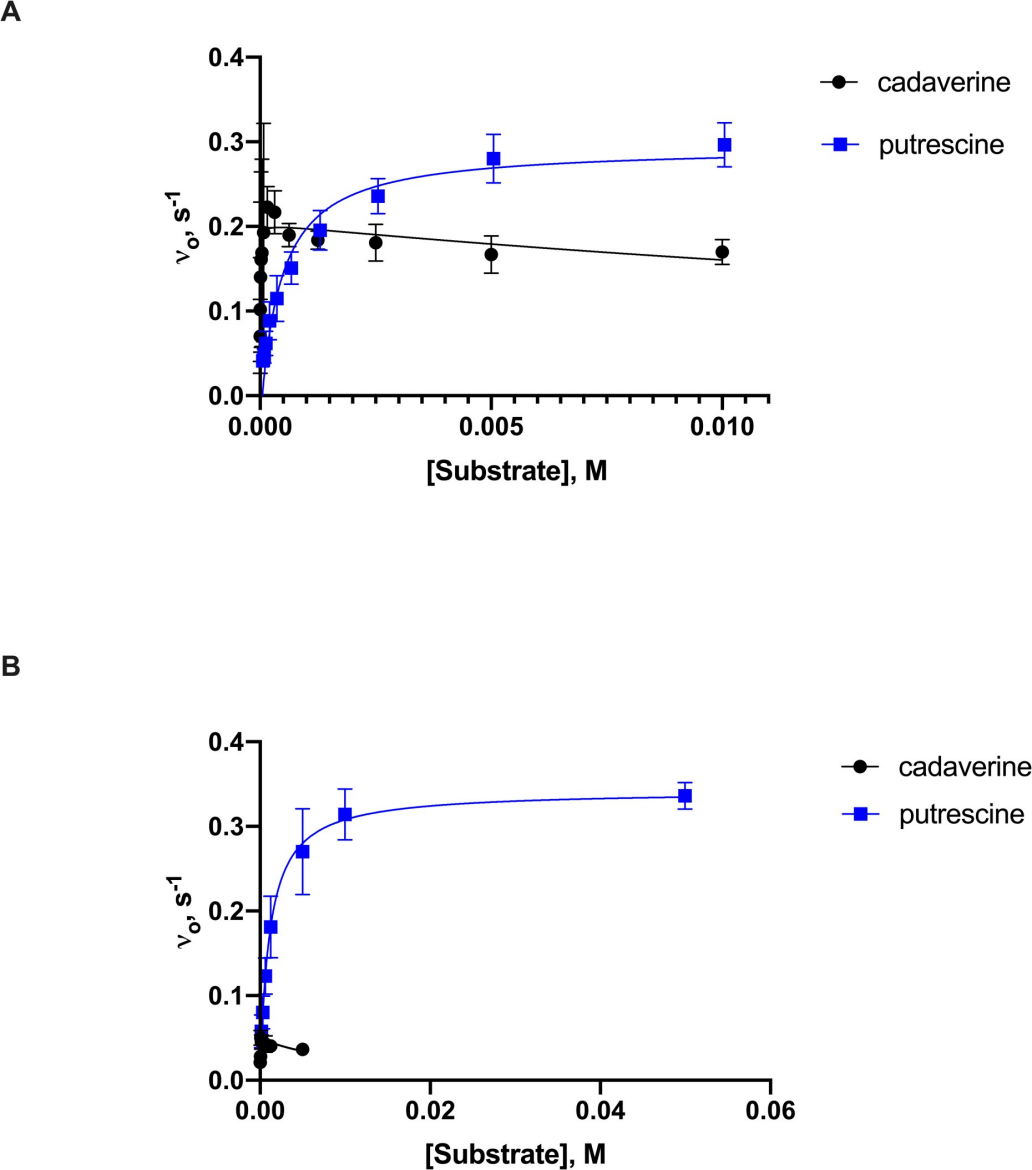
Representative steady-state kinetics of *Ss*DesB in oxygen consumption and product formation assays. A. Initial rate kinetic data obtained with cadaverine and putrescine in oxygen consumption assays. Kinetic data for putrescine assayed with *Ss*DesB, 0.05 mM FAD, and 0.7 mM NADPH. These data were obtained in triplicate and fit to the Haldane substrate inhibition (cadaverine) and Michaelis-Menten equations (putrescine), respectively. B. Initial rate data for cadaverine and putrescine assayed with *Ss*DesB, 0.05 mM FAD, and 0.7 mM NADH in product formation assays. These data were obtained in triplicate and fit to the Haldane substrate inhibition (cadaverine) and Michaelis-Menten equations (putrescine), respectively.

**Fig 4:**
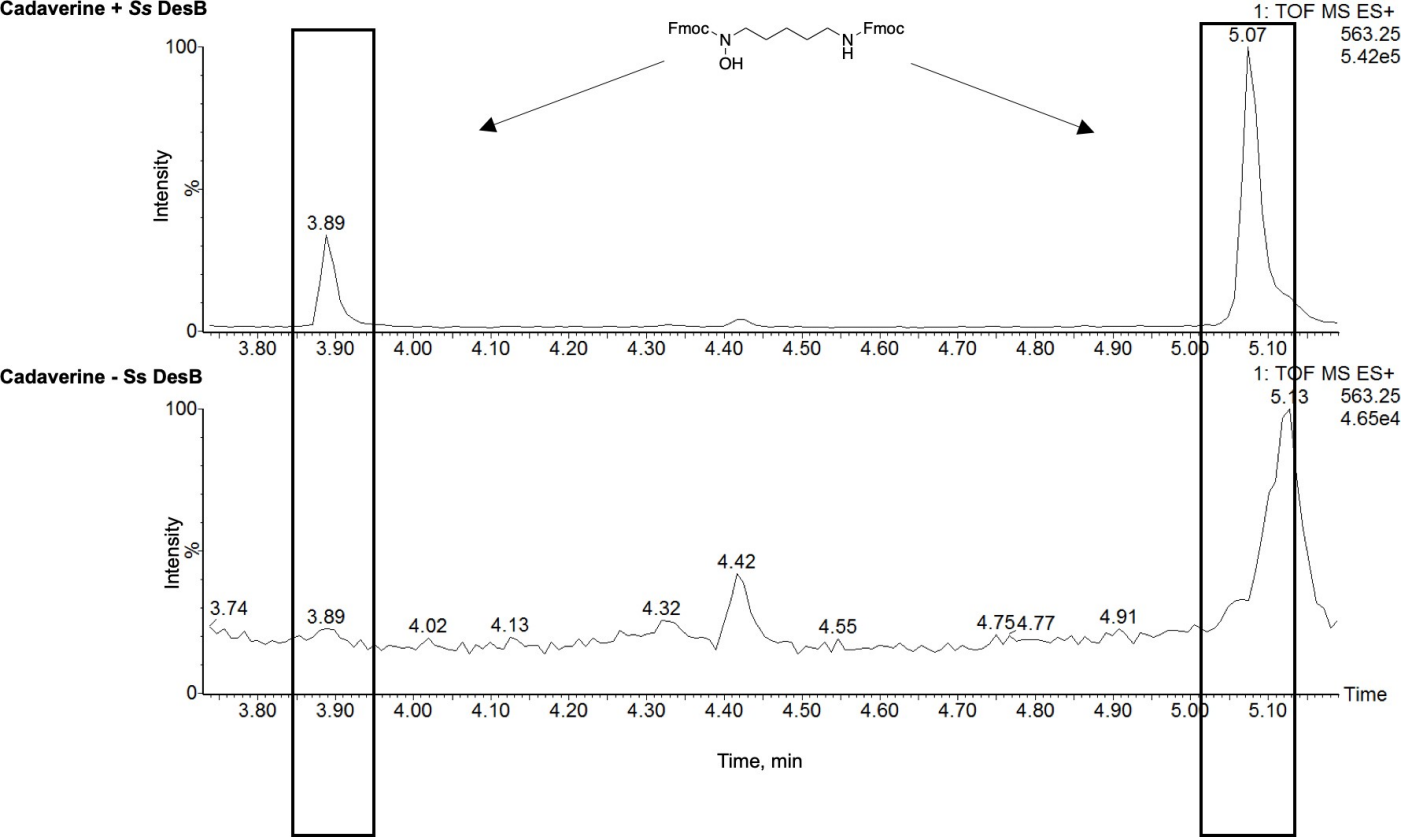
LC/MS detection of *N*-hydroxycadaverine (*m/z* 563.25). LC/MS select ion chromatograms of Fmoc-derivatized *N*-hydroxycadaverine in assays with and without *Ss*DesB. Diastereomers are observed.

Using *Ss*DesB without a hexahistidine tag at a constant concentration of NADPH, the enzyme *N*-hydroxylated cadaverine with an apparent *k*_cat_ of 0.048 ± 0.004 s^-1^ and catalytic efficiency of 2.5 ± 1.2 mM^-1^ s^-1^ (Table 3). However, the apparent *k*_cat_ value was approximately 6-fold lower than those determined in oxygen consumption and NADPH assays (Fig 3). *N*-hydroxylated product was confirmed by LC/MS in *Ss*DesB assays derivatized with Fmoc-Cl and not in assays without enzyme (Fig 4). At a constant NADPH concentration, *Ss*DesB also *N*-hydroxylated putrescine with a higher apparent *k*_cat_ of 0.034 ± 0.006 s^-1^ but lower catalytic efficiency of 0.31 ± 0.23 mM^-1^s^-1^, further indicating a preference for *N*-hydroxylating cadaverine over putrescine. The apparent K_M_ of cadaverine (31 μM) with *Ss*DesB was 35-fold lower than that of putrescine (1100 μM). When the NADH concentration was varied, the apparent *k*_cat_ value was ~2-fold higher than that when NADPH was varied, however the apparent K_M_ was ~4-fold larger, indicating a preference for binding NADPH over NADH. At high cadaverine and NAD(P)H concentrations, substrate inhibition was observed. To rule out product inhibition, we assayed *Ss*DesB in the presence of increasing concentrations of *N*-hydroxycadaverine and there was no decrease in enzyme activity (S10 Fig in S1 File). Thus, the initial rate data were fit to Eq 3 when inhibition was observed. Products were confirmed via LC/MS analysis of *Ss*DesB assays with cadaverine detected the generation of new compounds, such as *N*-hydroxycadaverine, after derivatization with Fmoc-Cl that were not present in control assays without enzyme (Fig 4).

**Table 3:** Steady-state kinetic parameters determined in product formation assays with *Ss*DesB and different substrates.

Substrate	*k*_cat_, s^-1^	*K*_m_, μM	*k*_cat_/*K*_m_, mM^-1^ s^-1^	*K*_*I*_, mM
Cadaverine	0.048 ± 0.004	19 ± 9	2.5 ± 1.2	27 ± 22
NADPH	0.034 ± 0.008	33 ± 24	1.0 ± 0.8	1.1 ± 0.6
Putrescine	0.34 ± 0.006	1100 ± 78	0.31 ± 0.23	
NADH	0.094 ± 0.018	130 ± 49	0.71 ± 0.26	1.0 ± 0.4

Initial rates measured with varied concentrations of cadaverine or putrescine (or 10 mM) in the presence of *Ss*DesB, 50 μM FAD, and 0.7 mM NAD(P)H (or varied concentrations). All measurements were made in triplicate.

### Substrate scope

**Fig 5:**
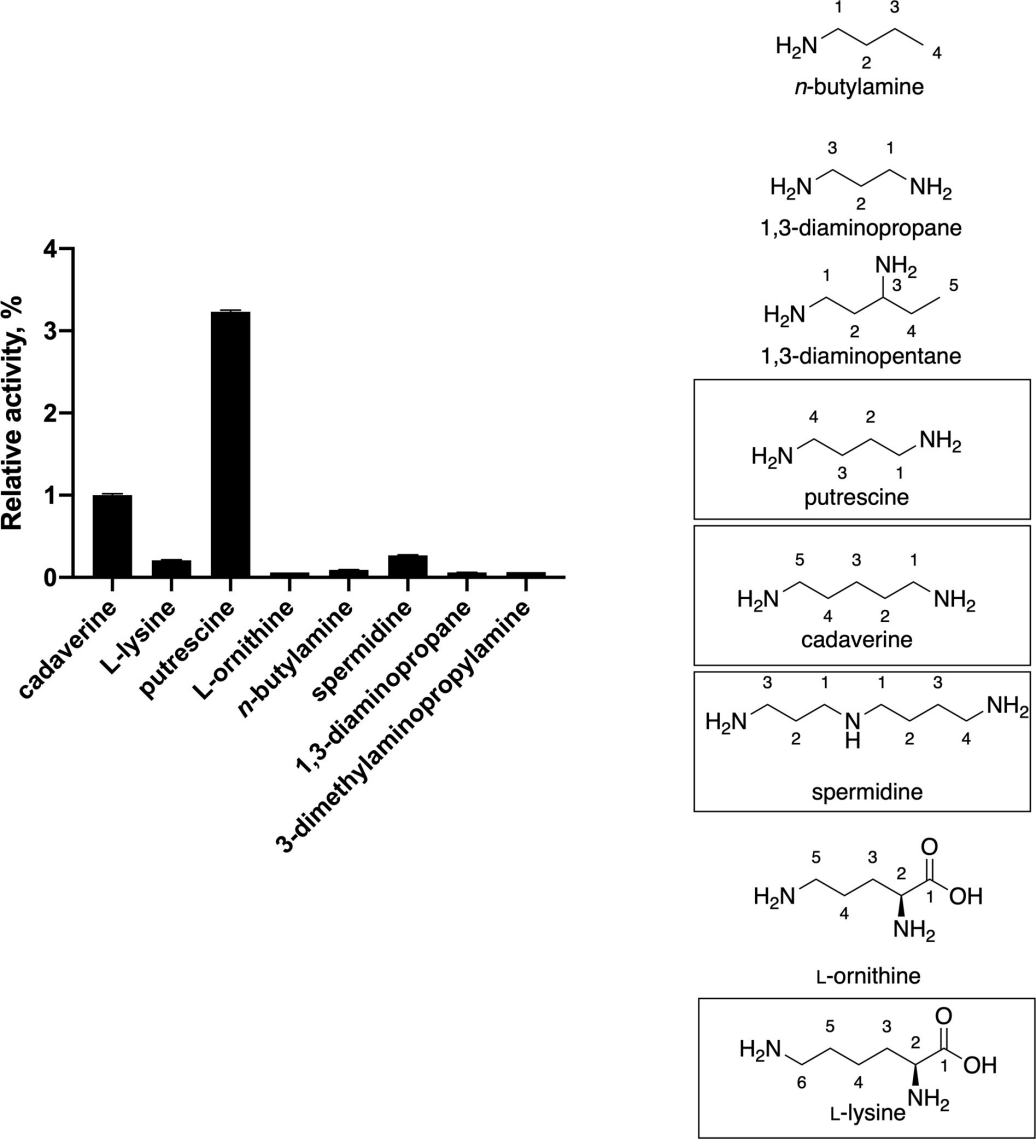
Relative *Ss*DesB activity with different substrates. Initial rates measured with 10 mM L-lysine, cadaverine, spermidine, putrescine, *n*-butylamine, L-ornithine, 1,3-diaminopropane, and 3-dimethylaminopropylamine in product formation assays in the presence of *Ss*DesB, 50 μM FAD, and 0.7 mM NADPH. All initial rates are relative to that of cadaverine. *n*-Butylamine, L-ornithine, 1,3-diaminopropane, and 3-dimethylaminopropylamine did not yield any Fmoc-derivatized *N*-hydroxylated products by LC/MS. All assays were performed in triplicate and molecular structures are shown with numbered carbon chains.

*Ss*DesB *N*-hydroxylated cadaverine, putrescine, spermidine, and L-lysine substrates (Fig 5). L-ornithine, 1,3-diaminopropane, and 3-dimethylaminopropylamine were not *N*-hydroxylated. The *m/z* values of these products are consistent with *N*-hydroxycadaverine ([M+H]^+^ *m/z* calcd. for C_35_H_34_N_2_O_5_H 563.25; found 562.2563), *N*-hydroxyputrescine ([M+H]^+^ *m/z* calcd. for C_34_H_32_N_2_O_5_H: 549.23; found 549.2389), *N*-hydroxylysine ([M+Na]^+^ calcd. for C_36_H_34_N_2_O_7_Na: 629.23; found 629.2264), and *N*-hydroxyspermidine ([M+H]^+^ *m/z* calcd. for C_37_H_39_N_3_O_5_H: 606.29; found 606.2968). See SI for extracted ion chromatograms and high resolution mass spectral data (S11–S15 Figs in S1 File). With the exception of assays with spermidine that yielded two stereoisomers of *N*-hydroxylated products, four stereoisomers of products were detected in all assays (S10–S13 and S15 Figs in S1 File). Assays were also performed with *Ss*DesB containing an *N*-terminal hexahistidine tag was assayed, and no *N*-hydroxylated products were detected in product formation assays containing *Ss*DesB with L-lysine. A slight difference in activity was only observed with L-lysine when it was assayed with the hexahistidine tagged *Ss*DesB, suggesting that the affinity tag interferes with the enzyme *N*-hydroxylating L-lysine.

### Structural analysis of *Ss*DesB

**Fig 6:**
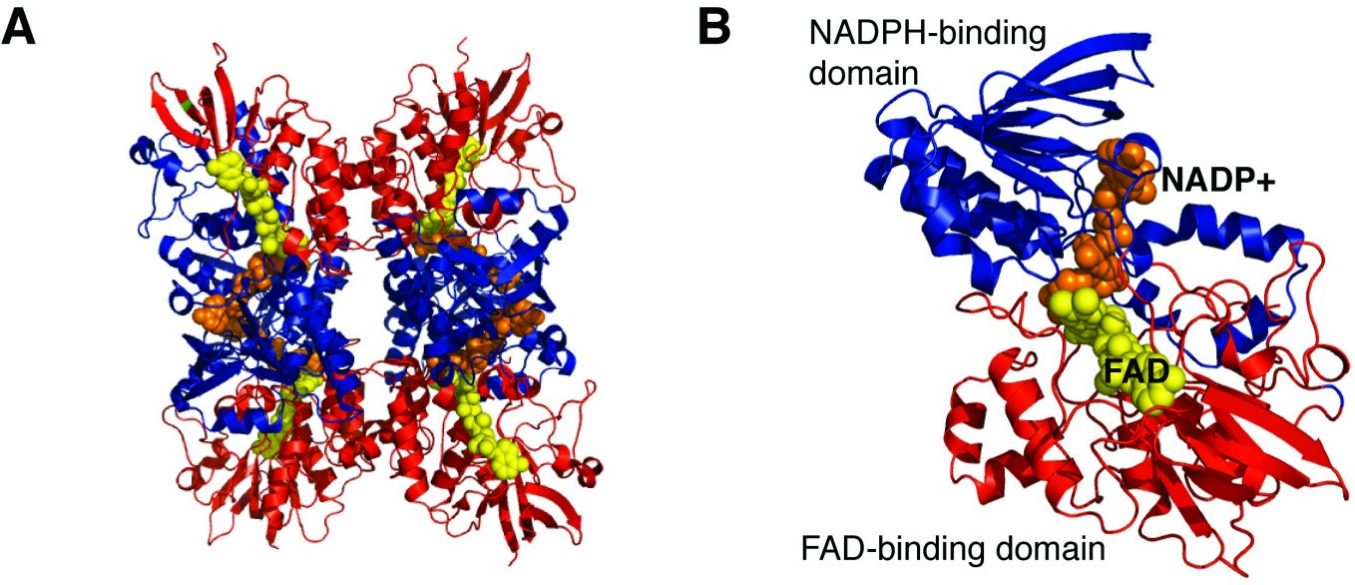
Overall structure of tetrameric *Ss*DesB in complex with NADP^+^. NAD^+^ and FAD-bound *Ss*DesB (PDB code: 6XBB). A) Tetrameric structure of *Ss*DesB complexed with NADP^+^ illustrating the FAD-binding domain (red-ribbons) and the NADPH-binding domain (blue-ribbons). The bound NADP^+^ molecule is shown in orange spheres and the FAD molecule in yellow spheres. B) A view of the *Ss*DesB protomer.

**Fig 7:**
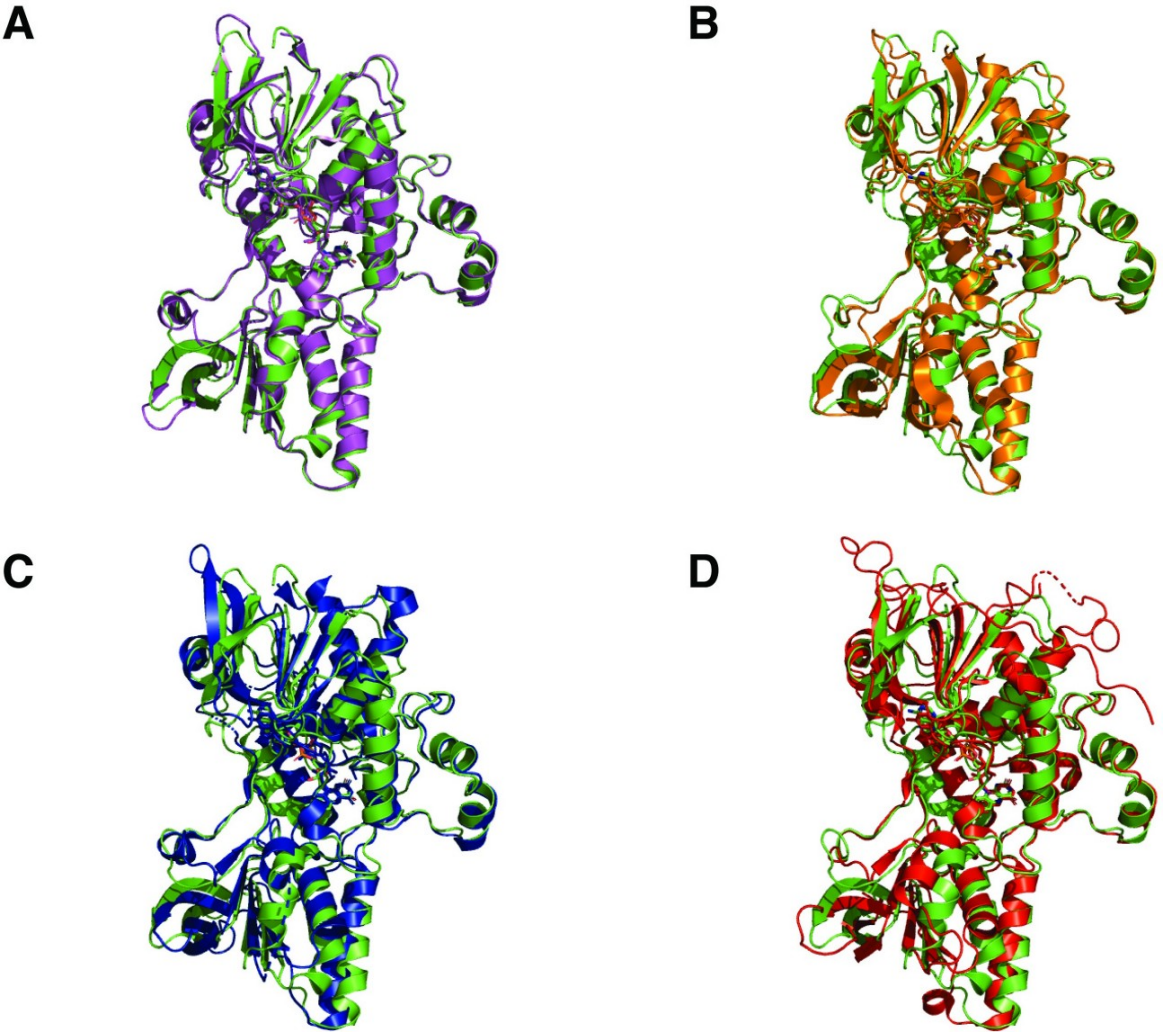
Structural comparison of *Ss*DesB and homologs. Superimposed crystal structures of chain A of *Ss*DesB (PDB code: 6XBC) with A) *E*. *amylovora* DfoA (PDB code: 5O8P), B) *Kutzneria* sp. 744 ornithine hydroxylase, Ktzl, (PDB code: 4TM1), C) *P*. *aeruginosa* ornithine hydroxylase, PvdA, (PDB code: 3S5W), and D) *A*. *fumigatus* ornithine hydroxylase, SidA, (PDB code: 5CKU).

Crystal structures of *Ss*DesB were obtained for the FAD-bound holoenzyme and the ternary complex with bound FAD and NADP^+^ at 2.86 Å and 2.37 Å, respectively. Data collection and processing statistics are presented in Table 4. *Ss*DesB crystallized as a homotetramer with each protomer consisting of two dinucleotide-binding Rossman-fold domains [40]: an FAD-binding domain (red) and an NADPH-binding domain (blue) (Fig 6). In both structures, each molecule in the asymmetric unit contained bound FAD and in the *Ss*DesB-FAD-NADP^+^ complex, a bound NADP^+^ molecule was clearly resolved in each protomer. Based on a query of the Protein Data Bank (PDB) using PDBefold [41, 42], the overall structure of the *Ss*DesB resembles those of previously determined crystal structures of the flavin-dependent monooxygenases *E*. *amylovora* DfoA (PDB entry 5O8P, r.m.s.d. 0.85 Å over 407 aligned residues) [43], the ornithine hydroxylase KtzI from *Kutzneria* sp. 744 (PDB entry 4TM1, r.m.s.d. 1.74 Å over 364 aligned residues) [5], the *P*. *aeruginosa* ornithine hydroxylase PvdA (PDB entry 3S5W, r.m.s.d 1.96 over 361 aligned residues) [44], and the *A*. *fumigatus* ornithine hydroxylase, SidA, (PDB entry 5CKU, r.m.s.d 2.08 Å over 362 aligned residues) (Fig 7 and S1 Table in S1 File).

**Table 4:** Crystallographic data collection and refinement statistics.

**Data collection statistics:**		
	***Ss*DesB-FAD**	***Ss*DesB-FAD-NADP**^**+**^
Program used:	*HKL*3000	*HKL*3000
Beamline	APS, SER-CAT, 22-ID	APS, SER-CAT, 22-BM
Wavelength (Å)	1.0000	1.0000
Space group	*P*2_1_	*P*2_1_
*a*, *b*, *c* (Å)	83.05, 151.17, 141.40	84.13, 153.43, 141.31
α, β, γ(°)	90, 91.58, 90	90, 92.44, 90
Resolution range*	50–2.86 (2.90–2.86)	50.0–2.37 (2.40–2.37)
Number of unique reflections measured	78957 (3917)	139487 (6942)
Completeness (%)	98.7 (98.5)	96.3 (96.3)
Redundancy	3.2 (3.3)	3.7 (3.7)
Mean I/σ(I)	15.4 (2.2)	14.2 (2.0)
*R*_*sym*_	0.066 (0.451)	0.089 (0.692)
*R*_*merge*_	0.043 (0.355)	0.059 (0.580)
*R*_*pim*_	0.042 (0.288)	0.053 (0.418)
CC ½	0.996 (0.860)	0.996 (0.729)
**Refinement statistics**		
Program used:	phenix.refine	phenix.refine
Resolution range (Å)	39.1–2.86	37.02–2.37
Number of reflections used in refinement	73546 (3617)	136497 (6786)
R/R_free_	0.199/0.258	0.189/0.240
No. of atoms		
Protein, chain A,B,C,D,E,F,G,H	3340/3340/3326/3326/3340/3333/3553/3340	3353/3340/3340/3346/3346/3340/3340/3370
FAD, chain A,B,C,D,E,F,G,H	53, all chains	53, all chains
NADP^+^	-	48, all chains
Water	-	1201
Average B factor (Å^2^)		
Protein, chain A,B,C,D,E,F,G,H	43.5/51.2/52.3/55.4/41.0/49.3/47.4/42.1	30.9/32.8/34.4/39.2/33.7/39.8/37.0/38.3
FAD, chain A,B,C,D,E,F,G,H	41.2/50.6/57.5/62.2/38.2/56.0/49.4/38.9	29.2/30.6/33.9/33.1/29.4/38.3/33.2/35.4
NADP^+^	-	31.5/33.4/35.6/38.3/36.2/41.0/38.5/38.8
Water	-	40.9
Root-mean-square- deviation from ideal		
Bond length (Å)	0.003	0.002
Bond angle (°)	0.6	0.5
**Ramachandran plot**		
Favored (%)	94.4	95.7
Allowed (%)	5.1	4.1
Outliers (%)	0.5	0.2
**Molprobity analysis**		
All atoms contact clash score	5.54 (100^th^ percentile)	3.1 (100^th^ percentile)
Molprobity score	1.66 (100^th^ percentile)	1.4 (100^th^ percentile)
PDB accession code	6XBC	6XBB

*Values in parenthesis are for the highest resolution shell

### FAD-bound enzyme

**Fig 8:**
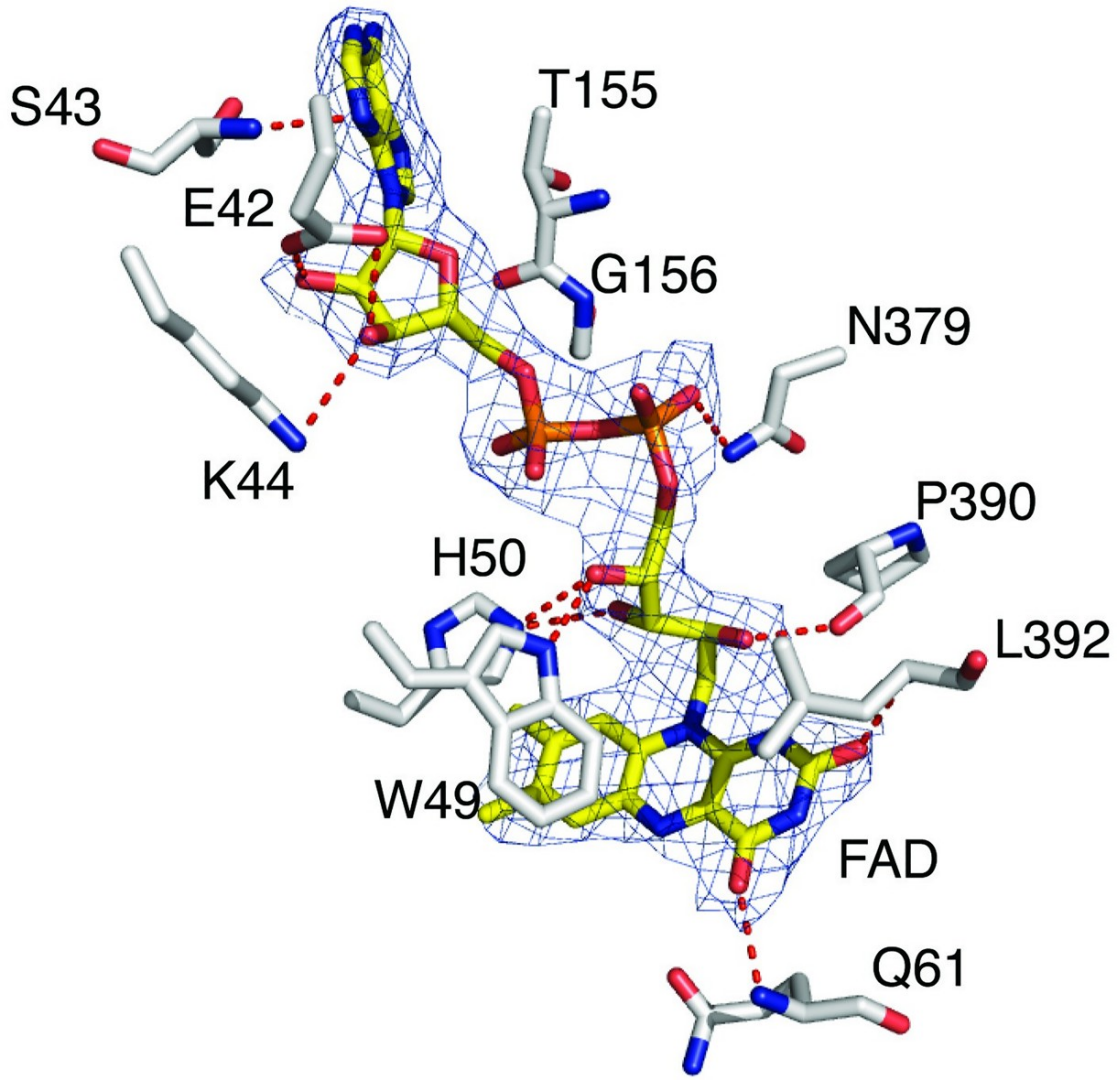
Active site environment of *Ss*DesB holoenzyme. View of the active site residues (carbon atoms in gray, nitrogen atoms in blue, and oxygen atoms in red) mediating FAD (carbon atoms in yellow, and phosphate atoms in orange) binding in the FAD-bound *Ss*DesB structure (PDB code: 6XBC). The fit of the FAD molecule to the final 2*F*_*o*_*-F*_*c*_ electron density map (blue mesh, 2.86 Å resolution, contoured at 1σ level) is shown.

In the *Ss*DesB-FAD complex structure, the FAD molecule is bound in an elongated conformation with the fully planar isoalloxazine ring positioned at the interface of the two dinucleotide-binding domains and a large area of the molecule, particularly the adenine dinucleotide portion, is exposed to solvent. During purification, after passage through the gel filtration column, fractions containing *Ss*DesB lost the deep yellow color, suggesting that FAD was lost during the process. For our crystallization efforts, we used concentrated protein eluted from an IMAC column in which the aliquots retained a deep yellow color. However, similar crystallization hits could also be obtained after adding exogenous FAD to the protein aliquots after passage through a size exclusion column and then concentrating the protein. Loss of FAD during purification was also reported with other homologs [3]. In the holoenzyme, FAD is held in the active site pocket primarily by hydrogen bonding interactions involving S43, E42, K44, H50, P390, and W49 along the flavin adenine nucleotide backbone while residues Q61 and L392 contribute direct hydrogen bonds to the planar isoalloxazine ring primarily via the backbone amide nitrogen atoms (Fig 8).

### FAD and NADP^+^-bound enzyme

**Fig 9:**
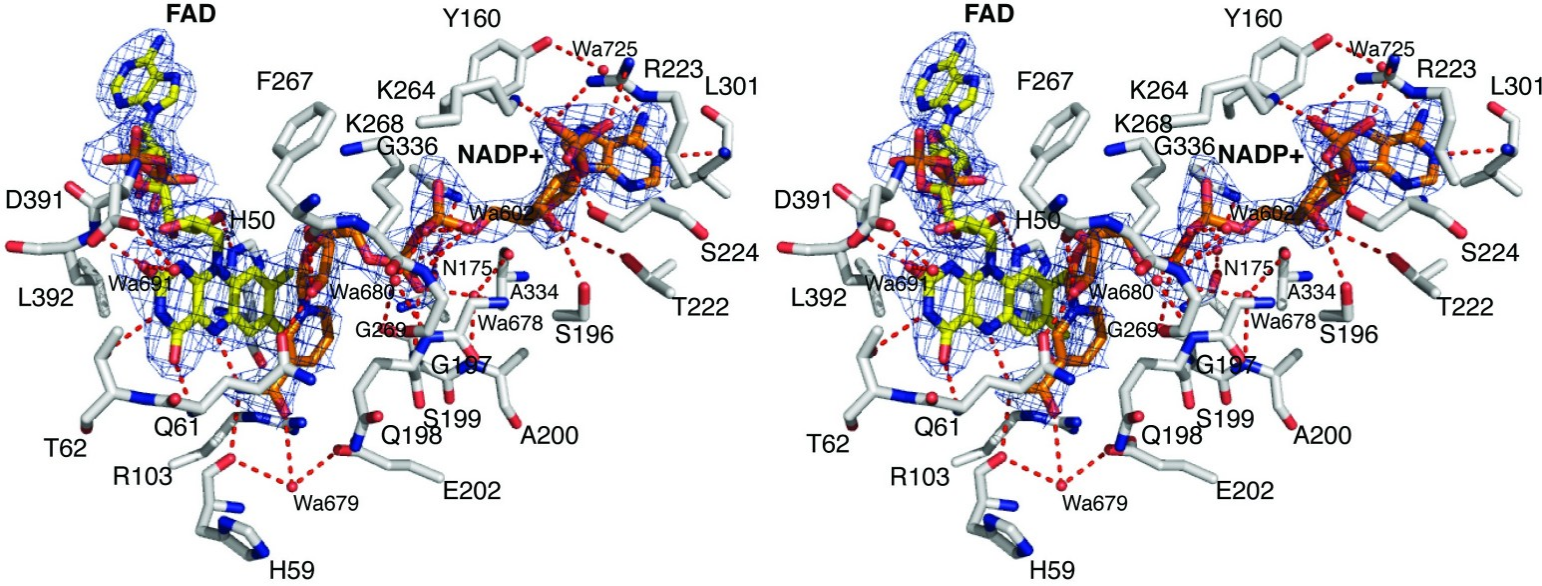
Active site environment of *Ss*DesB-NADP^+^ bound structure. Stereo-view of the active site of the *Ss*DesB-NADP^+^ bound structure (PDB code: 6XBB). The fit of the FAD molecule (carbon atoms in yellow, nitrogen atoms in blue, oxygen atoms in red, and phosphate atoms in orange) and the NADP^+^ molecule (carbon atoms in orange) to the final 2*F*_*o*_*-F*_*c*_ electron density map is shown (blue mesh, 2.37 Å resolution, contoured at 1σ level).

There are no significant global structural changes observed upon binding of the NADP^+^ molecule (r.m.s.d. = 0.26 over 2741 aligned atoms). The higher resolution 2.37 Å structure of the ternary complex shows that the NADP^+^ cofactor is held in the active site pocket in an elongated manner via several direct hydrogen bonds to *Ss*DesB and a network of water-mediated hydrogen bonding bridges between *Ss*DesB residues and NADP^+^ (Fig 9). The electron density is well-defined for both the FAD and NADP^+^ cofactors, including the nicotinamide ring, in all 8 protomers in the asymmetric unit. The nicotinamide ring is held in position by hydrogen bonding interactions between the nicotinamide N7N atom and main chain carbonyl oxygen atom of H59 and the N5 atom of the FAD isoalloxazine ring. The nicotinamide O7N atom participates in water-mediated hydrogen bonding interactions via water 679 to the E202 side chain and H50 main chain carbonyl oxygen atom. The side chain of Q61 also contributes to stabilizing interactions via a hydrogen bond between the side chain OE1 oxygen atom and the O2D ribose oxygen atom. The phosphate moiety of NADP^+^ is held in place by a salt bridge between the O2X oxygen and the side chain of K264, and the R232 side chain to the O2X and O1X oxygen atoms. In addition, there is a hydrogen bond between the O3X oxygen and the side chain of S224.

**Fig 10:**
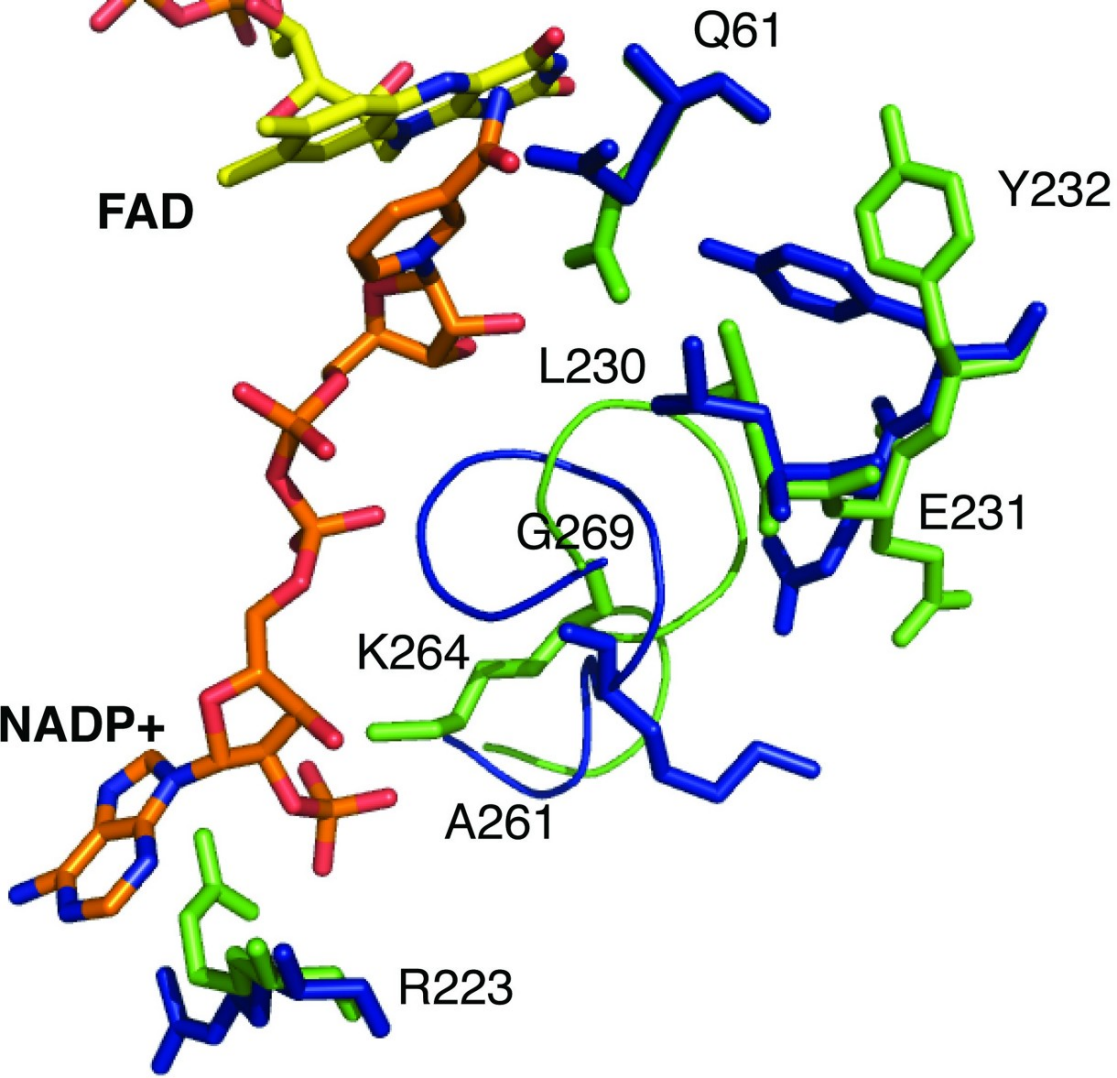
Structural changes upon NADP^+^ binding. View of the superimposed active sites of the *Ss*DesB holoenzyme (blue; PDB code: 6XBC) and *Ss*DesB-NADP^+^ bound (green; PDB code: 6XBB) structures highlighting the positional shift of residues in the active site upon NADP^+^ binding.

In comparison to the holoenzyme structure, upon NADP^+^ binding, several structural adjustments are observed among the residues surrounding the NADP^+^ binding pocket. At the interface of the FAD isoalloxazine ring and NADP^+^ nicotinamide ring, the side chain of Q61 shifts away from the FAD isoalloxazine to accommodate the binding of the nicotinamide ring of NADP^+^, which in turn, induces a shift in the rotamer positions of the nearby L230, E231, and Y232 side chains (Fig 10). Additionally, the D391 side chain is now engaged in a water-mediated hydrogen bond bridge between the side chain OD2 oxygen atom and the isoalloxazine ring O2 atom via water 691 (Fig 9). The side chain of T62 is also hydrogen bonded to the isoalloxazine ring N3 nitrogen atom (Fig 9). A shift in the conformation of the loop consisting of residues 261–269 is observed which avails space for NADP^+^ binding and also properly positions the side chain of K264 to engage in an ionic interaction with the phosphate moiety of NADP^+^. The side orientation of R223 also shifts and is now properly positioned for an ionic interaction with the NADP^+^ phosphate and is also stacked against the terminal adenine moiety.

## Discussion

Alkyl diamine *N*-hydroxylases are class B flavin monooxygenases that catalyze the *N*-hydroxylation of diamines using NADH or NADPH as their redox partners. These NMOs play an essential role in committing diamines to the biosynthesis of hydroxamate siderophores, which are used to sequester iron for microorganisms, including several human pathogens. Furthermore, they are used in the industrial fermentation of pharmaceuticals, such as desferrioxamine B, and are currently being explored as biocatalysts, as they accept complex substrates and introduce the unique N-O functionality in a single step, which can be further derivatized [45]. However, they are not as well-studied as the L-lysine and L-ornithine NMOs, particularly those involved in the biosynthesis of nocobactin in *N*. *farcinica* [3] and ferrichrome in *A*. *fumigatus* [4], respectively. Here we report the structural and kinetic characterization of *Ss*DesB, a cadaverine *N*-hydroxylase from *S*. *sviceus*.

**Fig 11:**
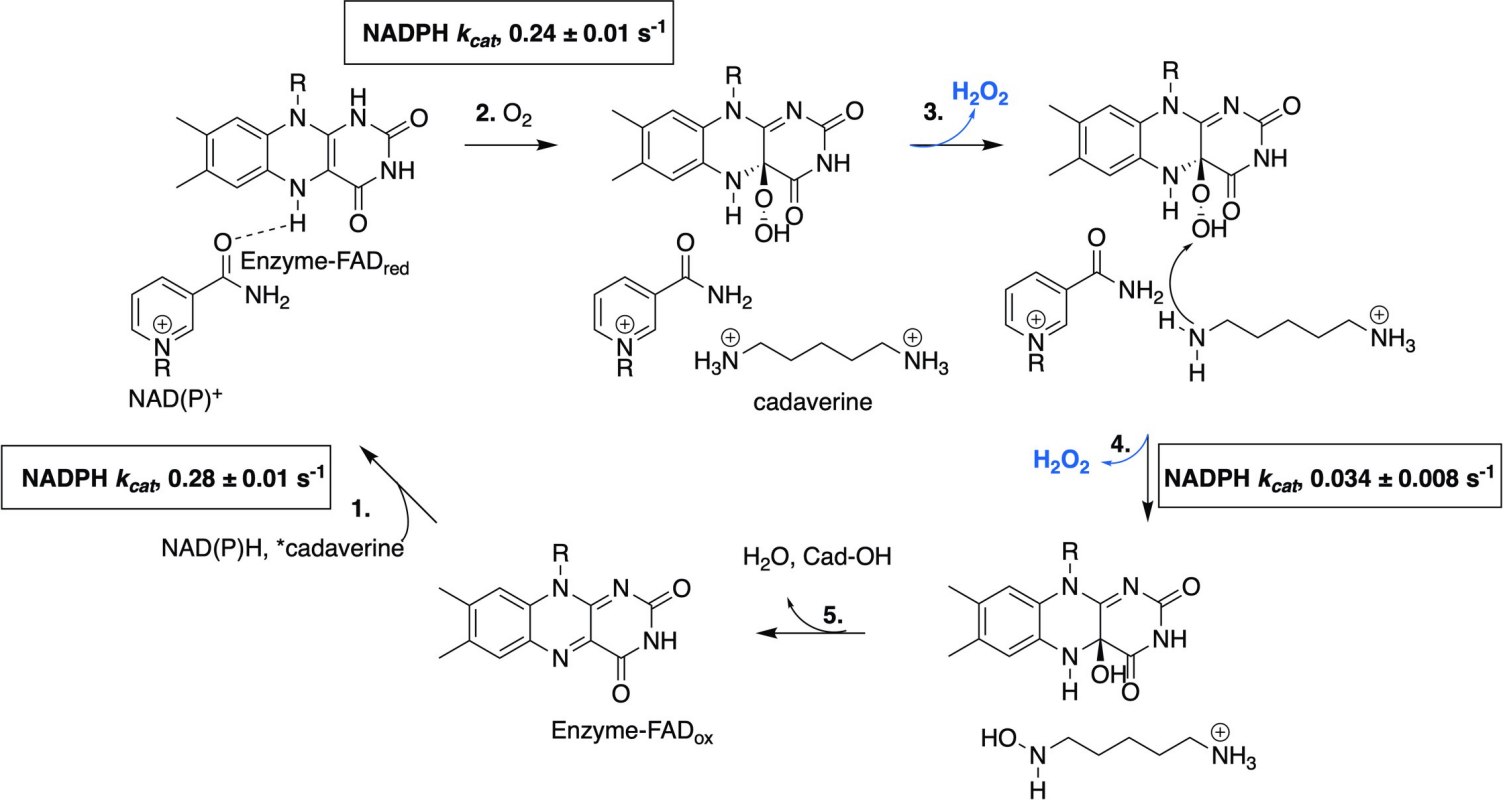
General mechanism for Class B flavin-dependent monooxygenases. The NADPH oxidation, oxygen consumption, and *N*-hydroxylation steps are represented by steps 1, 2, and 4, respectively. The loss of peroxide is indicated in blue and the apparent *k*_*cat*_ values for steady-state kinetic assays with varied NADPH concentrations are shown. *, the substrate is either added before or after the reduction of molecular oxygen.

The general mechanism proposed for class B flavin-dependent monooxygenases (Fig 11) commences with an oxidized FAD being reduced by the C4-pro-R position of NAD(P)H nicotinamide to the *N*5 position of the flavin isoalloxazine ring to produce FADH^-^ (FAD_red_). Then molecular oxygen adds to FAD_red_ to form an activated C4a-(hydro)peroxyflavin intermediate that undergoes nucleophilic attack by the primary amine of the bound substrate, which could bind before or after the reduction of molecular oxygen. NADP^+^ typically remains bound to prevent the quenching of the C4a-(hydro)peroxyflavin species [46, 47]. Once the hydroxylated product is formed, the oxidized FAD is regenerated by the loss of water and the products dissociate, completing the catalytic cycle. Based on the kinetic analysis of the NADPH oxidation, O_2_ consumption, and *N*-hydroxylation steps, *Ss*DesB appears to have a mechanism consistent with the proposed mechanism for a Class B flavin-dependent monooxygenase. However, Fig 2 shows that *Ss*DesB consumes oxygen when incubated with NADPH in the absence of substrate, suggesting that the substrate may not be required to be bound to *Ss*DesB for FAD to be reduced (step 1 in Fig 11), which is not uncommon for *N*-hydroxylases [46, 48].

While the apparent *k*_*cat*_ values for the initial NADPH oxidation and oxygen consumption steps for *Ss*DesB were of the same order of magnitude, there was a ten-fold difference in the apparent *k*_*cat*_ values of those steps to that of the *N*-hydroxylation step. The significant decrease in the apparent *k*_*cat*_ among those three steps indicates the uncoupling of the reaction. The detection of peroxide in *Ss*DesB assays with cadaverine further supports this notion, as not all molecular oxygen is converted into *N*-hydroxylated product. Other *N*-hydroxylases have been reported to catalyze uncoupled reactions, such as NbtG (*Nocardia farcinica*) [49], MbsG (*Mycobacterium smegmatis*) [49], PvdA [48], and GorA [1].

Unlike many amino acid *N*-hydroxylases with K_M_ values ranging from 0.3 to 4 mM, the apparent K_M_ values for cadaverine, NADPH, and NADH were significantly lower, approaching the limit of detection of the multistep product formation assay. The apparent *k*_cat_ of *Ss*DesB with cadaverine was at least 10-fold less than the published *k*_cat_ values of other amino acid *N*-hydroxylases [50]. *Ss*DesB also exhibited higher apparent *k*_cat_ values with putrescine, which were the same order of magnitude in product formation and oxygen consumption assays, suggesting the putrescine substrate stabilizes the C4a-(hydro)peroxyflavin intermediate. However, cadaverine is the preferred substrate based on its higher apparent catalytic efficiency. We are aware that the apparent K_M_ values for cadaverine and putrescine in product formation assays were slightly higher than those determined in the other assays, which is likely due to greater error associated with the multistep product formation assay.

The difference in the magnitudes of the apparent K_M_ values for cadaverine and putrescine, which only differ by one methylene group, likely reflects the physiological relevance of these polyamines in *S*. *sviceus*. For example, in *S*. *putrefaciens*, desferrioxamine B is synthesized once putrescine has been depleted [51]. The more than 20-fold difference between the apparent K_M_ values of cadaverine and putrescine with *Ss*DesB could represent a way to manage iron acquisition in *S*. *sviceus*. The *S*. *sviceus* genome contains genes to transport putrescine into the cell; thus, putrescine could be used as a *Ss*DesB substrate to produce *N*-hydroxyputrescine and other putrescine-derived hydroxamate siderophores under certain growth conditions.

Based on the *N*-hydroxylation of cadaverine and putrescine, *Ss*DesB has broader substrate and cofactor specificity than other class B flavin-dependent monooxygenases supporting our initial hypothesis. *Ss*DesB exhibited similar activity to GorA [1] and PubA [26], accepting both NADPH and NADH cofactors as well as multiple diamine substrates. However, a higher apparent catalytic efficiency was determined with NADPH, indicating a preference for NADPH over NADH. A preference for NADPH was also reported for GorA, in which the relative amount of hydroxylated product made with NADPH was significantly higher than with NADH, which produced too little product for quantification in assays containing 150 μM of either cofactor. Similar to GorA, *Ss*DesB also exhibits higher activity with putrescine followed by cadaverine [1]. However, the authors did not report steady-state kinetic data over a wide enough range of cadaverine concentrations to confirm that putrescine is the preferred GorA substrate [1]. While *Ss*DesB exhibits higher activity with putrescine at concentrations above its apparent K_M_, cadaverine is the preferred substrate based on its low micromolar K_M_, increasing its apparent catalytic efficiency.

Molecules containing alkyl chains with less than four methylene groups or branched alkyl amines were not *N*-hydroxylated by *Ss*DesB. Spermidine and L-lysine were accepted to a lesser degree, respectively, indicating that *Ss*DesB can tolerate longer alkyl diamines as well as a carboxyl group. LC/MS detected at least four stereoisomers of these *N*-hydroxylated products with the exception of spermidine, which produced two stereoisomers likely due to the *N*-hydroxylation of the longer alkyl chains, as the amines with 3-carbon alkyl chains were not *N*-hydroxylated. The spermidine substrate is more readily *N*-hydroxylated than L-lysine (Fig 5) and may form more stable hydrogen bonding interactions with its additional nitrogen atoms. Interestingly, assays with *Ss*DesB containing an *N*-terminal hexahistidine tag did not *N*-hydroxylate lysine, whereas *Ss*DesB did without a hexahistidine tag, suggesting that additional residues near the *N*-terminus prevent the *N*-hydroxylation of amino acid substrates (S16 Fig in S1 File).

**Fig 12:**
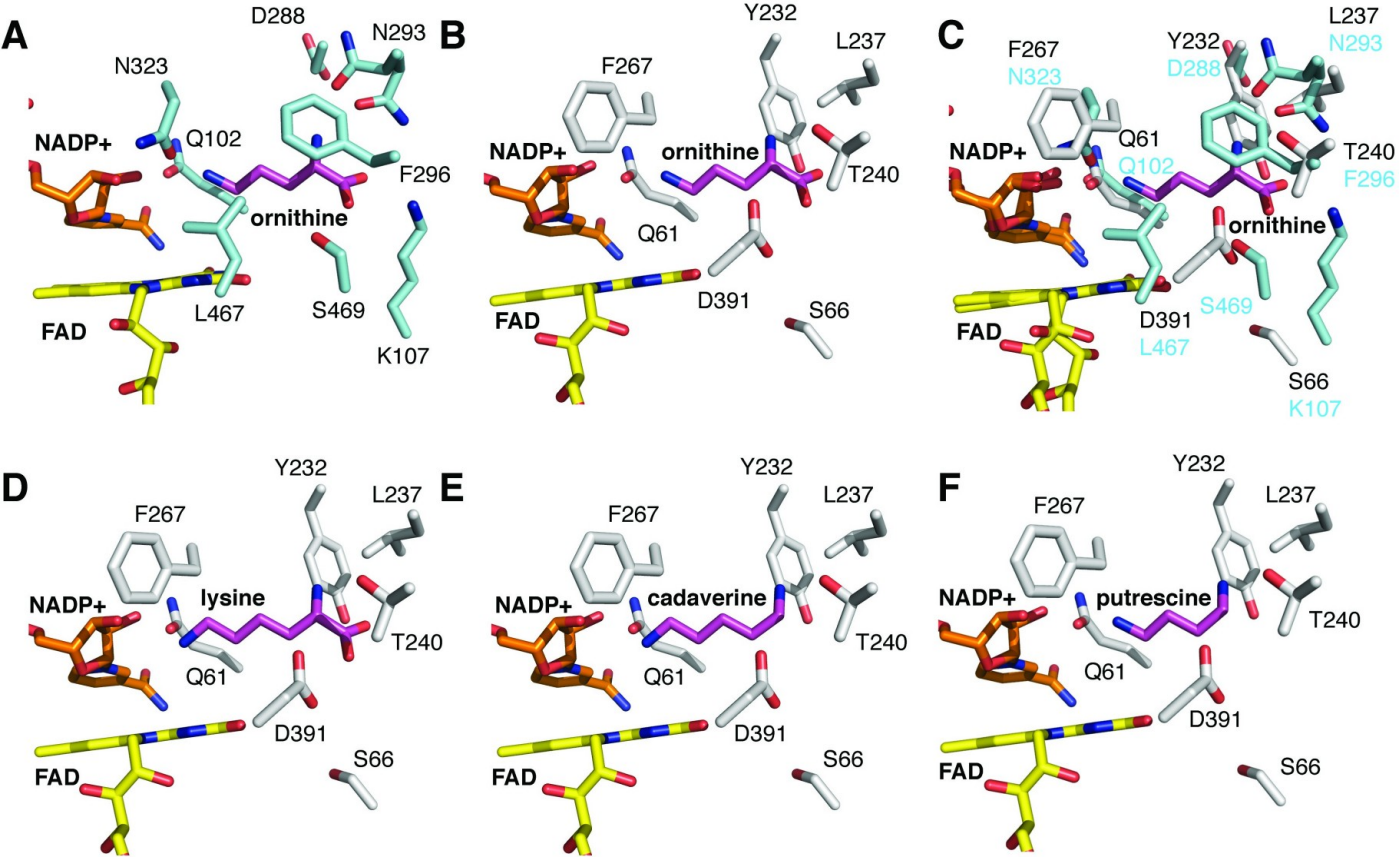
Structural analysis of substrate specificity. A. Active site structure of SidA bound to NADP^+^ and ornithine (PDB code: 4b63). B. Model of ornithine binding to the active site of *Ss*DesB obtained by superimposing the coordinates of *Ss*DesB (PDB code: 6XBB) onto the coordinates of SidA bound to ornithine (PDB code: 4b63). C. Comparison of the active site environment between *Ss*DesB and SidA. D. Modeling of lysine binding to *Ss*DesB. E. Modeling of cadaverine binding to *Ss*DesB. F. Modeling of putrescine binding to *Ss*DesB.

The structural basis for *Ss*DesB *N*-hydroxylation was explored by comparing the structure of *Ss*DesB bound to NADP^+^ with experimental crystal structures of the SidA homolog with bound substrate. Similar to other NMO structures [5], *Ss*DesB is a homotetramer with its active site within a subunit at the interface of three domains, including Rossman-type FAD and NAD(P)H binding domains (Fig 6). The *Ss*DesB active site appears to be more solvent exposed, which may contribute to its uncoupled mechanism as a lower amount of FAD copurified with the enzyme compared to that of other *N*-hydroxylases (75% flavin in NbtG [3] and 50–60% flavin in SidA [4]) in which FAD is buried and inaccessible by solvent. While *Ss*DesB accepts a broad range of substrates, such as cadaverine and putrescine, it does not *N*-hydroxylate L-ornithine. To gain structural insight into the substrate specificity of *Ss*DesB, we superimposed the coordinates of the *Ss*DesB-NADP^+^ complex onto its SidA homolog, which accepts L-ornithine as a substrate, crystallized with NADP^+^ and L-ornithine (PDB code: 4b63) [52] (Fig 12). Fig 12A shows that SidA binds L-ornithine through several hydrogen bonds involving N323, N293, S469, and K107 residues (these residues are conserved in the PvdA and KtzI homologs); however, *Ss*DesB lacks the equivalent residues (Fig 12B and 12C). In particular, *Ss*DesB lacks the equivalent K107 residue, which would hydrogen bond to the carboxylate moiety of L-ornithine and contribute a stabilizing positive charge, anchoring the substrate. Additionally, *Ss*DesB contains a nonpolar L237 residue (N293 in SidA), eliminating the possibility of hydrogen bonding interactions at this position with the carboxylate oxygen and *δ*-carbon primary amine of L-ornithine. In SidA, N323 hydrogen bonds with the ⍺-carbon primary amine; however, *Ss*DesB contains a nonpolar F267 residue in the equivalent position (Fig 12C). Furthermore, a clashing negative charge provided by *Ss*DesB D391 (L467 in SidA, L404 in KtzI and L408 in PvdA) would likely repel the negative charge of the L-ornithine carboxylate group. These highlighted structural features provide insight into why *Ss*DesB does not accept L-ornithine as a substrate.

Given that *Ss*DesB accepts L-lysine, cadaverine, and putrescine as substrates, we modeled the position of these substrates into the *Ss*DesB active site by superimposing the *Ss*DesB coordinates onto available crystal structures from homologs with bound substrates (Fig 12). The position of L-lysine in the *Ss*DesB active site was modeled by overlaying the *Ss*DesB coordinates onto those of the SidA-NADP^+^- L-lysine complex structure (Fig 12D, PDB code: 4b64) and the position of L-ornithine was based on the superimposed coordinates with the SidA-NADP^+^- L-ornithine structure (Fig 12B, PDB code: 4b63), while the position of cadaverine (Fig 12E) and putrescine (Fig 12F) was modeled based on the removal of atoms from the L-lysine substrate in PDB entry 4b64 and L-ornithine substrate in PDB entry 4b63, respectively. While exact hydrogen bonding interactions are difficult to predict based on modeling alone, we can suggest that given proper positioning of cadaverine, the side chain of D391 may hydrogen bond with the ε-carbon amine and Q61 is within hydrogen bonding distance to the ⍺-carbon amine (Fig 12). Similar interactions could possibly be observed with putrescine, which is one methylene shorter than cadaverine, given proper positioning of the substrate. However, given putrescine’s lower apparent K_M_ with *Ss*DesB, these interactions may be weaker due to the shorter chain length. Although we observed low activity with L-lysine, we could not fully rationalize the binding of L-lysine based on our modeling studies. If the side chain of D391 moved away from the carboxylate moiety of L-lysine, then the nearby T240 side chain could hydrogen bond with L-lysine. However, experimental X-ray crystal structures and additional site-directed mutagenesis studies are needed to fully characterize the structural basis for the binding of *Ss*DesB substrates and identify key catalytic residues.

We also examined the X-ray crystal structure to understand the preference of *Ss*DesB for NADPH over NADH. The ornithine *N*-hydroxylase from *P*. *aeruginosa*, PvdA [5, 48], only uses NADPH as the electron donor during the oxidation of substrates. In the 1.9 Å resolution crystal structure of PvdA with bound NADP^+^ and ornithine (PDB code: 3S5w), there is no electron density available to sufficiently model the nicotinamide ring, suggesting that flexibility may be required for catalysis [5]. However, in *Ss*DesB, the electron density for the nicotinamide ring of NADP^+^ is fully resolved in all molecules in the asymmetric unit. The specificity of PvdA for NADPH is determined by two residues, R240 and S210 [5]. R240 forms two hydrogen bonds with the 2’-phosphate on the adenine ribose and the S286 residue also forms a hydrogen bond to the phosphate. In contrast, *Ss*DesB has an R223 residue, which is equivalent to R240 in PvdA, but lacks the equivalent serine residue (S3 Fig in S1 File). Instead, a positively charge lysine, K268, is positioned away from the 2’-phosphate, which may reduce the specificity of *Ss*DesB for NADPH (Fig 9).

## Conclusions

The present study is the first kinetic and structural characterization of *Ss*DesB, providing insight into the biosynthesis of desferrioxamine and derivatives. The kinetic and structural data revealed the broader substrate scope of alkyl amine *N*-hydroxylases compared to the more selective ornithine and lysine *N*-hydroxylases. Furthermore, an uncoupled mechanism was observed when *Ss*DesB was assayed with cadaverine and not with putrescine. Understanding the structural and catalytic basis for activity can lead to the development of biocatalysts and inhibitors that target homologs in human pathogens. Future studies include the kinetic and structural characterization of mutated *Ss*DesB residues to identify key catalytic residues, understand why the enzyme-catalyzed reaction is uncoupled with cadaverine, and expand the *Ss*DesB substrate scope to produce structurally diverse hydroxamate siderophores and *N*-hydroxylated molecules.

## Supporting information

S1 File (DOCX)

S1 Raw images (PDF)
