# Supplementary material for: Characterization of a broadly specific cadaverine N-hydroxylase involved in desferrioxamine B biosynthesis in Streptomyces sviceus
Source: PLoS One. 2021 Mar 30;16(3):e0248385. doi: 10.1371/journal.pone.0248385 (PMC8009421; doi:10.1371/journal.pone.0248385)
Supplement: S1 File — (DOCX) [file pone.0248385.s001.docx]

**Characterization of a broadly specific cadaverine *N*-hydroxylase involved in desferrioxamine B biosynthesis in *Streptomyces sviceus***

Lesley-Ann Giddings, George T. Lountos, Kang Woo Kim, Matthew Brockley, Danielle Needle, Scott Cherry, Joseph E. Tropea, David S. Waugh

*Table of Contents*

|  | Page |
| --- | --- |
| Figure S1. Sequence similarity network | 3 |
| Figure S2. Phylogenetic tree of characterized *N*-hydroxylases | 4 |
| Figure S3. Sequence alignment of *Ss*DesB and homologs. | 5 |
| Figure S4. Purification summary of *Ss*DesB with FAD | 6 |
| Figure S5. QuantiChrome peroxide formation assay results | 7 |
| Figure S6. Initial rate kinetic data obtained with varied concentrations of NAD(P)H in NAD(P)H oxidation assays. | 8 |
| Figure S7. Initial rate kinetic data obtained with varied concentrations of cadaverine and putrescine in NADH oxidation assays. | 9 |
| Figure S8. Initial rate kinetic data obtained with varied concentrations of NADPH in oxygen consumption assays. | 9 |
| Figure S9. Representative steady-state kinetic data obtained with varied concentrations of NAD(P)H in product formation assays. | 10 |
| Figure S10. Initial rate kinetic data of SsDesB with varied concentrations of cadaverine in the presence of *N*-hydroxycadaverine in product formation assays. | 11 |
| Figure S11. LC/MS traces showing broader substrate specificity. | 11 |
| Figure S12. LC/MS *m/z* data of *Ss*DesB Fmoc-derivatized cadaverine and *N*-hydroxylated product. | 12 |
| Figure S13. LC/MS *m/z* data of *Ss*DesB Fmoc-derivatized putrescine and *N*-hydroxylated product. | 13 |
| Figure S14. LC/MS *m/z* data of *Ss*DesB Fmoc-derivatized spermidine and *N*-hydroxylated product. | 14 |
| Figure S15. LC/MS *m/z* data of *Ss*DesB Fmoc-derivatized L-lysine and *N*-hydroxylated product. | 15 |
| Figure S16. Relative rates of *N*-hydroxylation determined in product formation assays with *Ss*DesB with an *N*-terminal hexahistidine tag. | 16 |
| Table S1. Structure Alignments of *Ss*DesB with homologs | 17 |
| References | 18 |

**Sequence similarity network and phylogenetic analysis**

Using NCBI pBLAST [40], 5000 sequences with an e-value of 2e-98 were selected to be similar to *Ss*DesB (GenBank ID: WP_007382224.1). The Enzyme Function Initiative-Enzyme Similarity Tool (EFI-EST) [41] was then used to select proteins with no more than 50% sequence identity (alignment score of 137) to create a similarity network using default parameters. Cytoscape v3.7.0 [42] was used to visualize the network using the preferred layout setting.


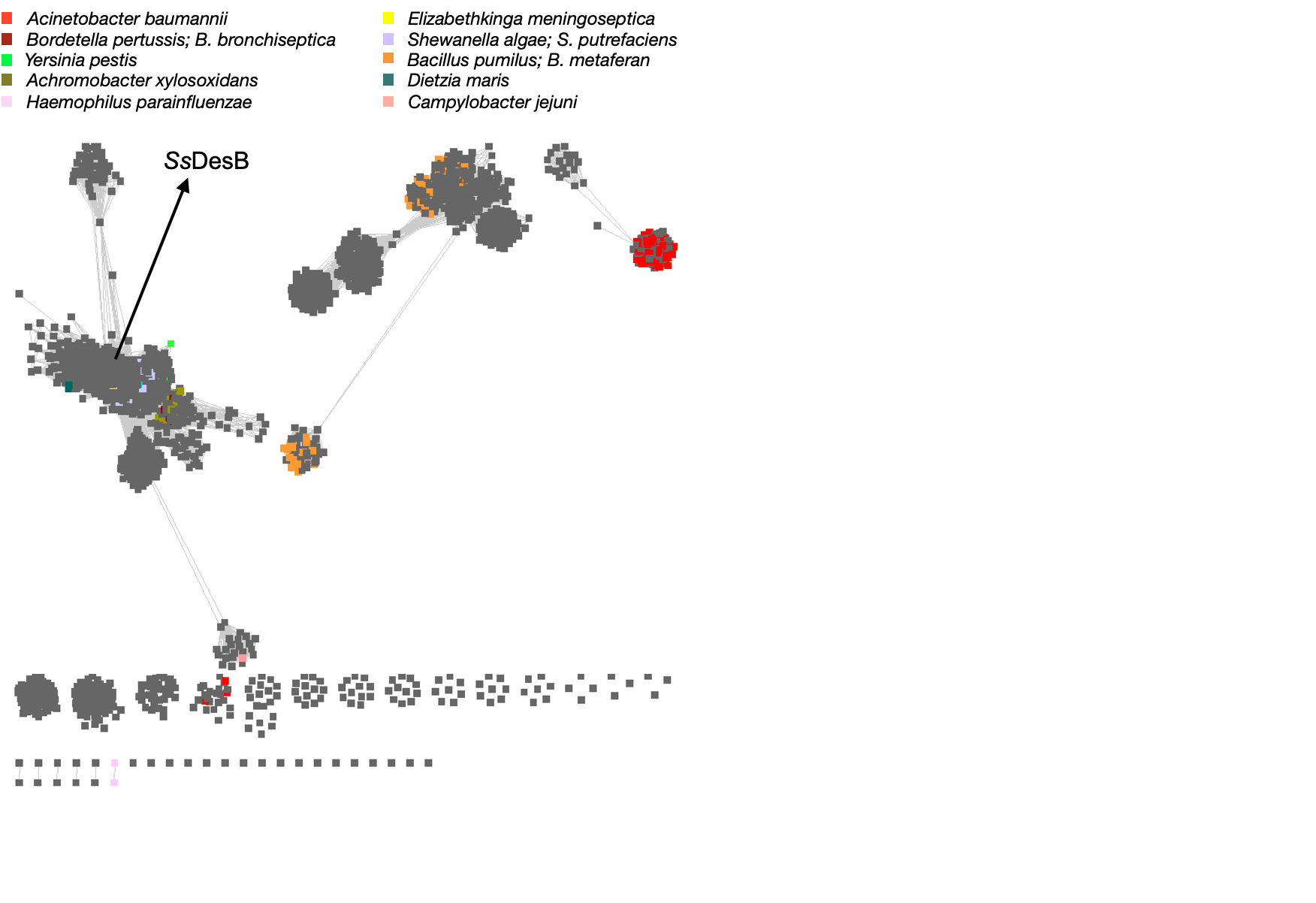


**Figure S1.**  A sequence similarity network of 5,001 nodes (sequences) that are similar to *Ss*DesB, each sequence is >40% in sequence identity. Edges represent an E-value threshold of 2e-98. Nodes associated with clinical pathogens are colored and labeled as shown in the figure. Clusters with less than 5 sequences have been removed.

**
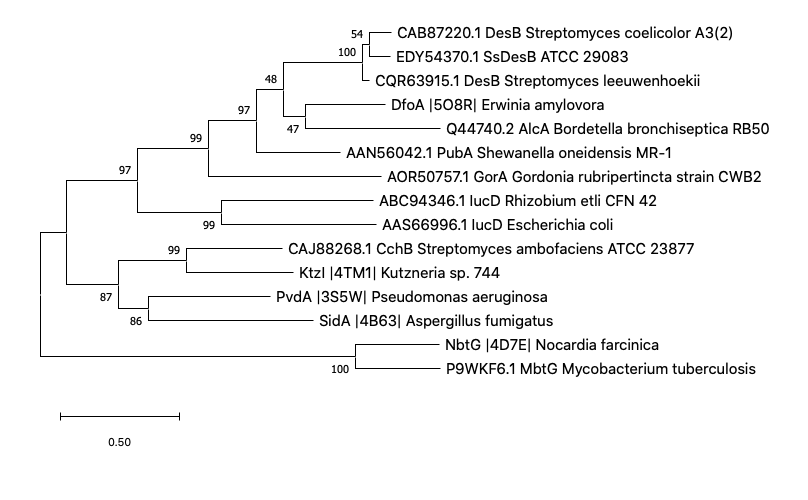
**

**Figure S2.** Relationship between *Ss*DesB and other characterized *N*-hydroxylases. Maximum likelihood phylogenetic tree of characterized *N*-hydroxylases with bootstrapping values made using MEGA7.


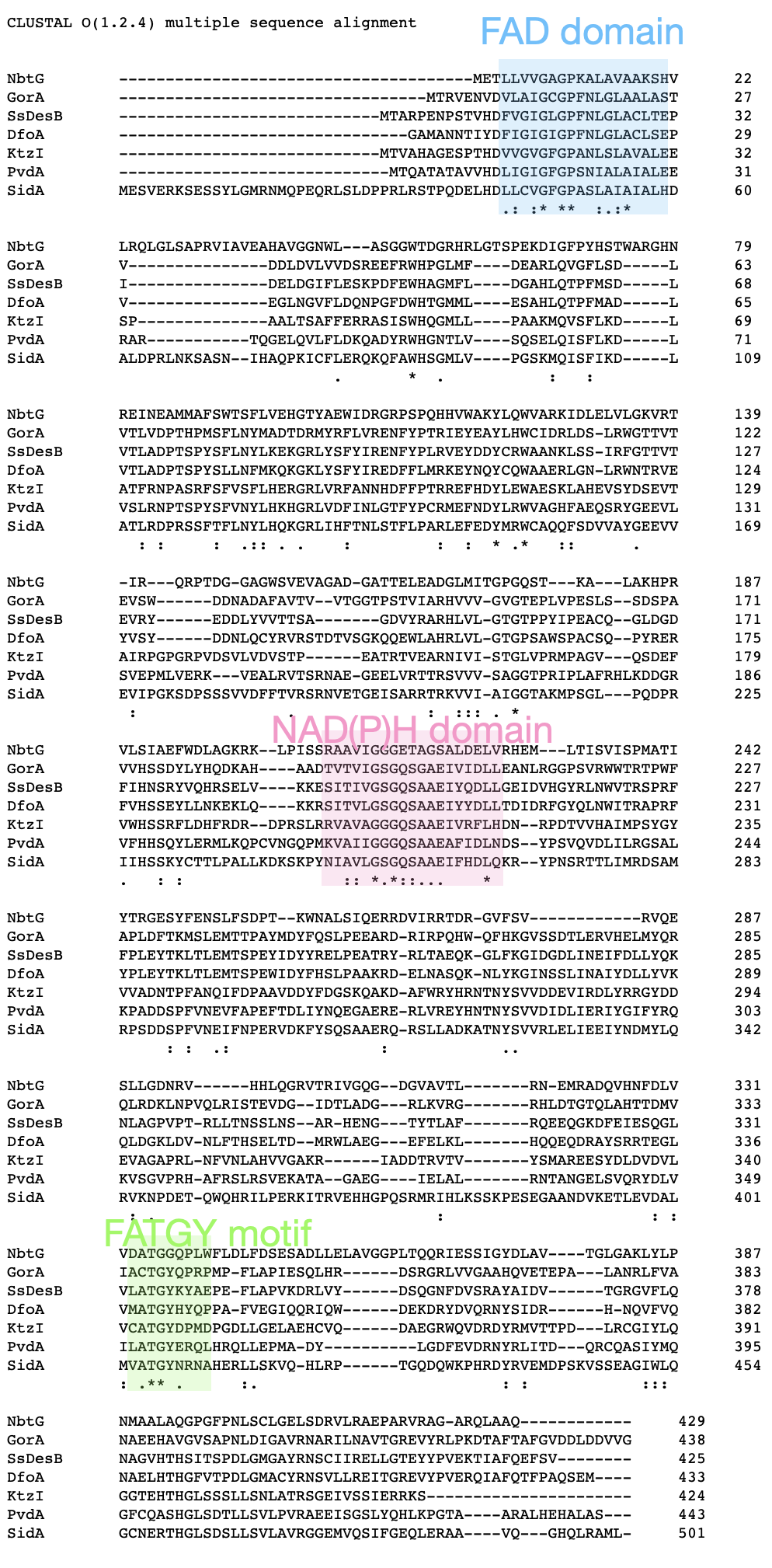


**Figure S3.** **Sequence alignment of *Ss*DesB and homologs**. Clustal Omega alignment (<http://www.ebi.ac.uk/Tools/msa/clustalo/>) [2] of cadaverine-N hydroxylases, *Ss*DesB and DfoA; putrescine *N*-hydroxylase, GorA; lysine *N*6-hydroxylase, PvdA; ornithine *N*5-hydroxylases, KtzI and SidA. Blue, pink, and green boxes indicate conserved FAD, NAD(P)H, and FATGY motifs.


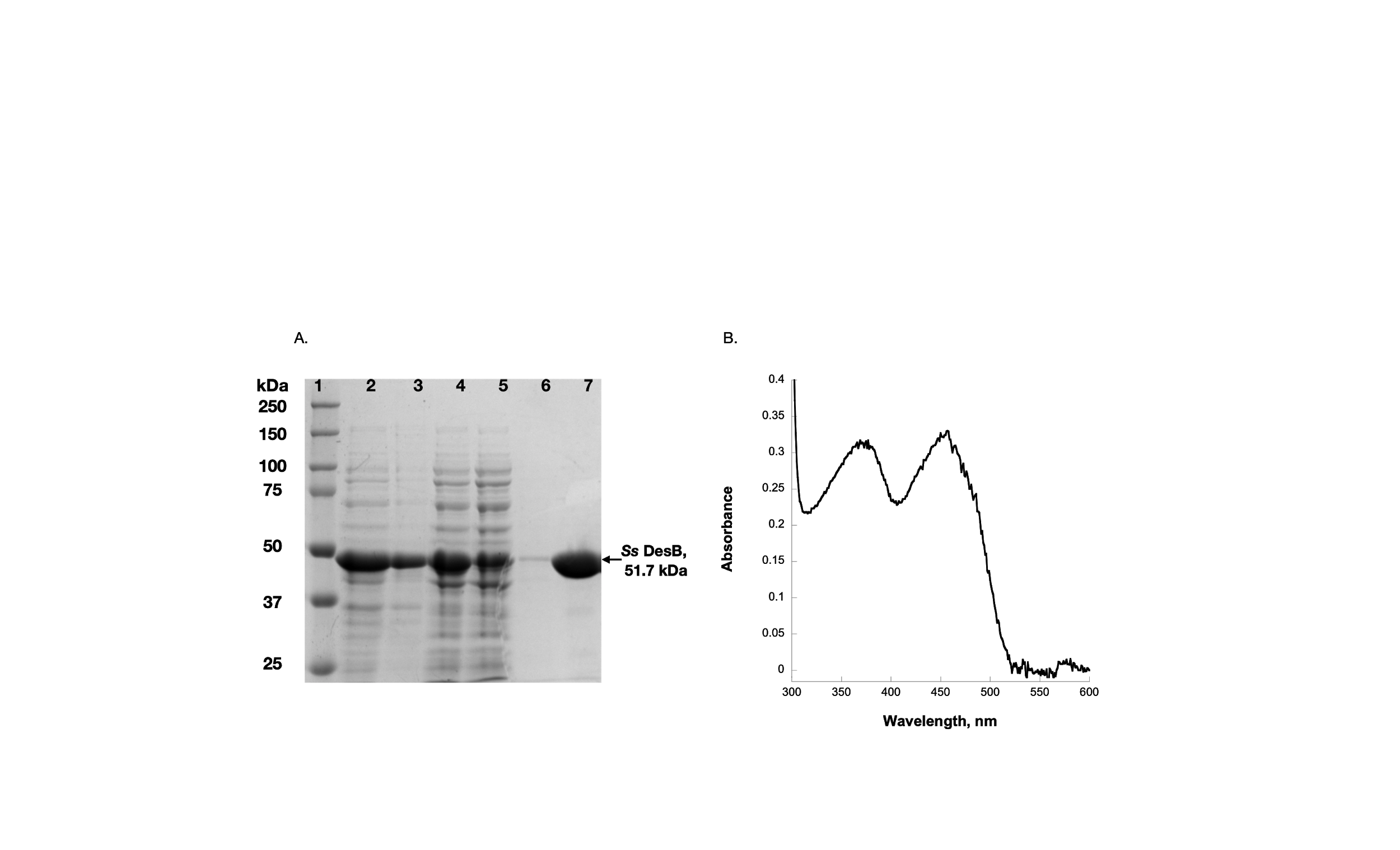


**Figure S4. Summary of *Ss*DesB purification with FAD.** A) SDS-PAGE of *Ss*DesB purification. Lane 1, molecular mass marker; lane 2, crude lysate; lane 3, pellet; lane 4, supernatant; lane 5, flow-through; lane 6, wash; lane 7, elution. B) UV-visible spectrum of FAD bound to purified *Ss*DesB.

A) Calibration curve 1 quenched at t = 30 s

1. Calibration curve 1 quenched at t = 72 s

1. Photo of 96-well plate showing assays with and without SsDesB.


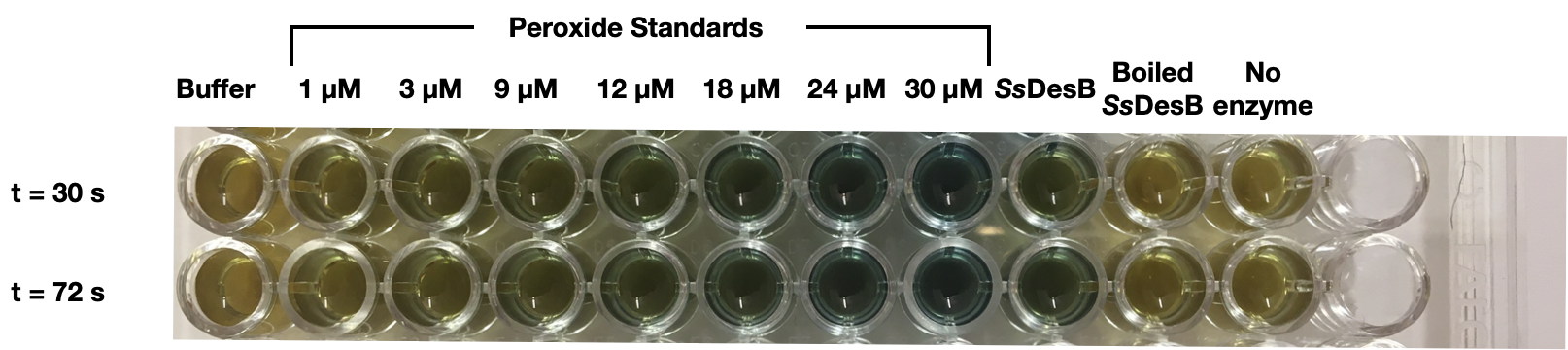


**Figure S5. Colorimetric QuantiChrome peroxide formation assay.** A) Calibration curve of peroxide detected in assays when question 30 s after assay was initiated. B) Calibration curve of peroxide detected in assays when question 72 s after assay was initiated. C) Peroxide was detected in assays with *Ss*DesB (2.12 μM) incubated for 30 s and 72 s with cadaverine (10 mM), NADPH (0.7 mM), and FAD (50 μM) before being quenched with peroxide detection reagents.


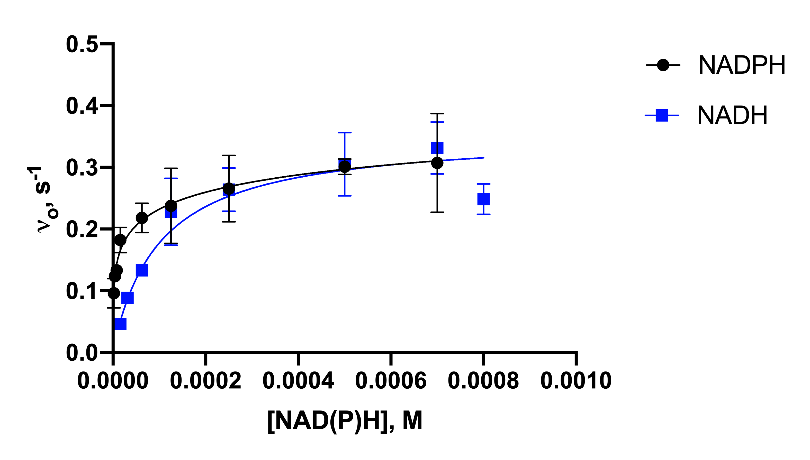


**Figure S6. Initial rate kinetic data obtained with NAD(P)H in NAD(P)H oxidation assays.** Kinetic data for NADPH and NADH assayed with *Ss*DesB, 0.05 mM FAD, and 10 mM cadaverine. These data were obtained in triplicate and fit to the Michaelis-Menten (NADPH) and the Haldane substrate inhibition (NADH) equations, respectively.

**Figure S7. Initial rate kinetic data obtained with cadaverine and putrescine in NADH oxidation assays.** Kinetic data for varying concentrations of cadaverine (black) or putrescine (blue) assayed with *Ss*DesB, 0.05 mM FAD, and 0.7 mM NADH. These data were obtained in triplicate and fit to the Haldane substrate inhibition (cadaverine) and Michaelis-Menten equations (putrescine), respectively.

**
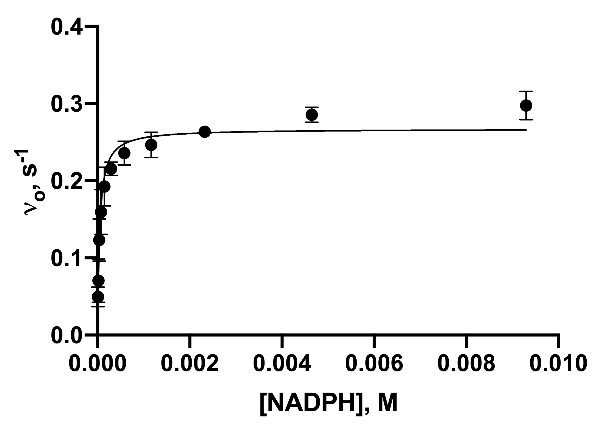
**

**Figure S8. Initial rate kinetic data obtained with NADPH in oxygen consumption assays.** Kinetic data for varying concentrations of NADPH assayed with *Ss*DesB, 0.05 mM FAD, and 10 mM cadaverine. These data were obtained in triplicate and fit to the Michaelis-Menten equation.


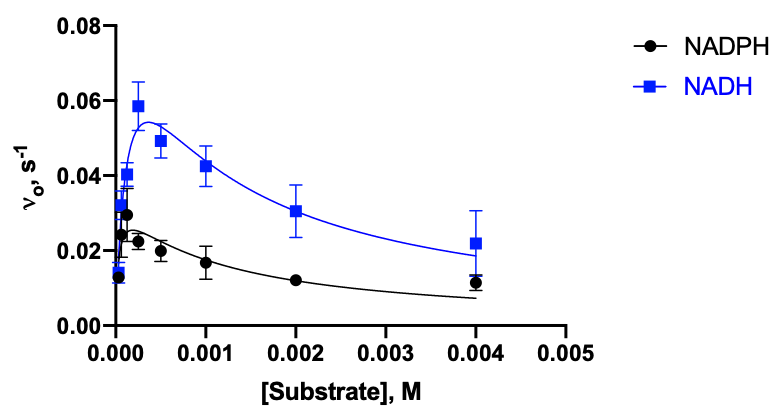


**Figure S9. Representative steady-state kinetic data obtained in product formation assays.** Kinetic data for varying concentrations of NADPH (black) and NADH (blue) assayed with *Ss*DesB, 0.05 mM FAD, and 10 mM cadaverine. These data were obtained in triplicate and fit to the Haldane substrate inhibition.


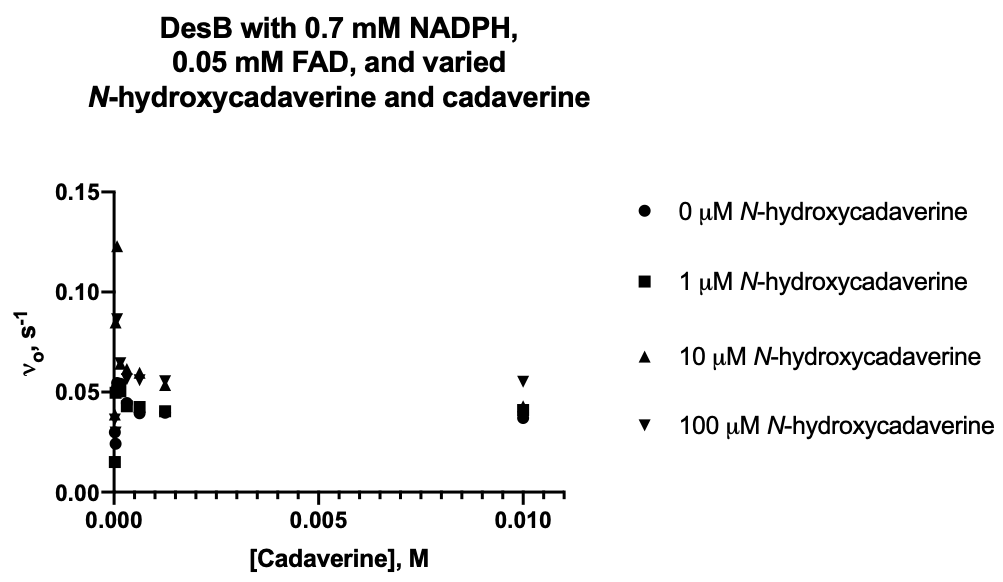


**Figure S10.** **Initial rate kinetic data of SsDesB with cadaverine in the presence of *N*-hydroxycadaverine in product formation assays.** Kinetic data for various concentrations of *N*-hydroxycadaverine product added to *Ss*DesB assays containing varying concentrations of cadaverine, 0.05 mM FAD, and 0.7 mM NADPH. These data were obtained in triplicate.


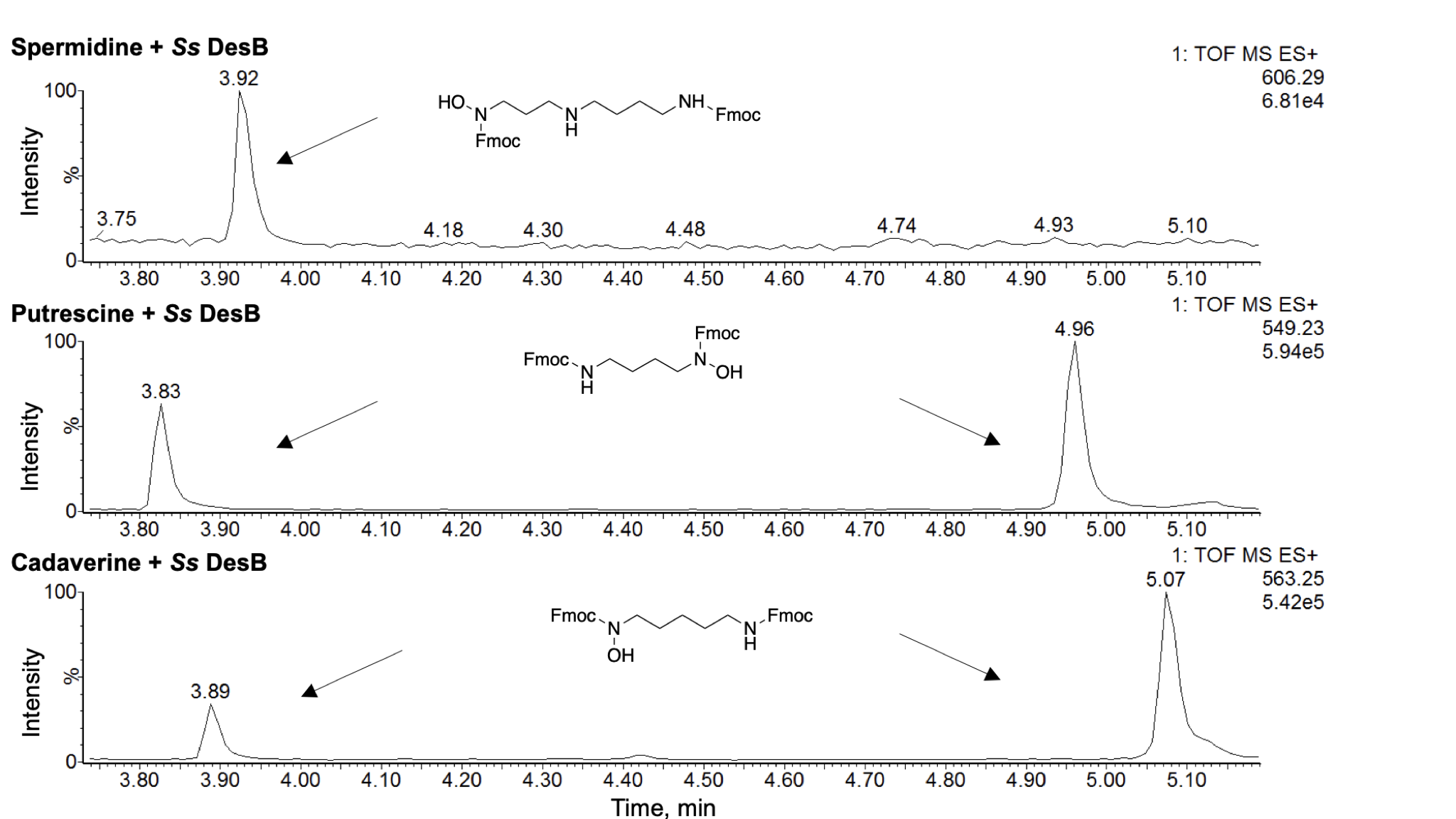


**Figure S11. UPLC-HRMS traces showing broader substrate specificity.** UPLC-HRMS select ion chromatograms of various Fmoc-derivatized N-hydroxylated products of assays with and without Ss DesB. Diastereomers are observed with putrescine and cadaverine (Only two out of four are shown). A product could not be detected under these conditions from assays with lysine.

A
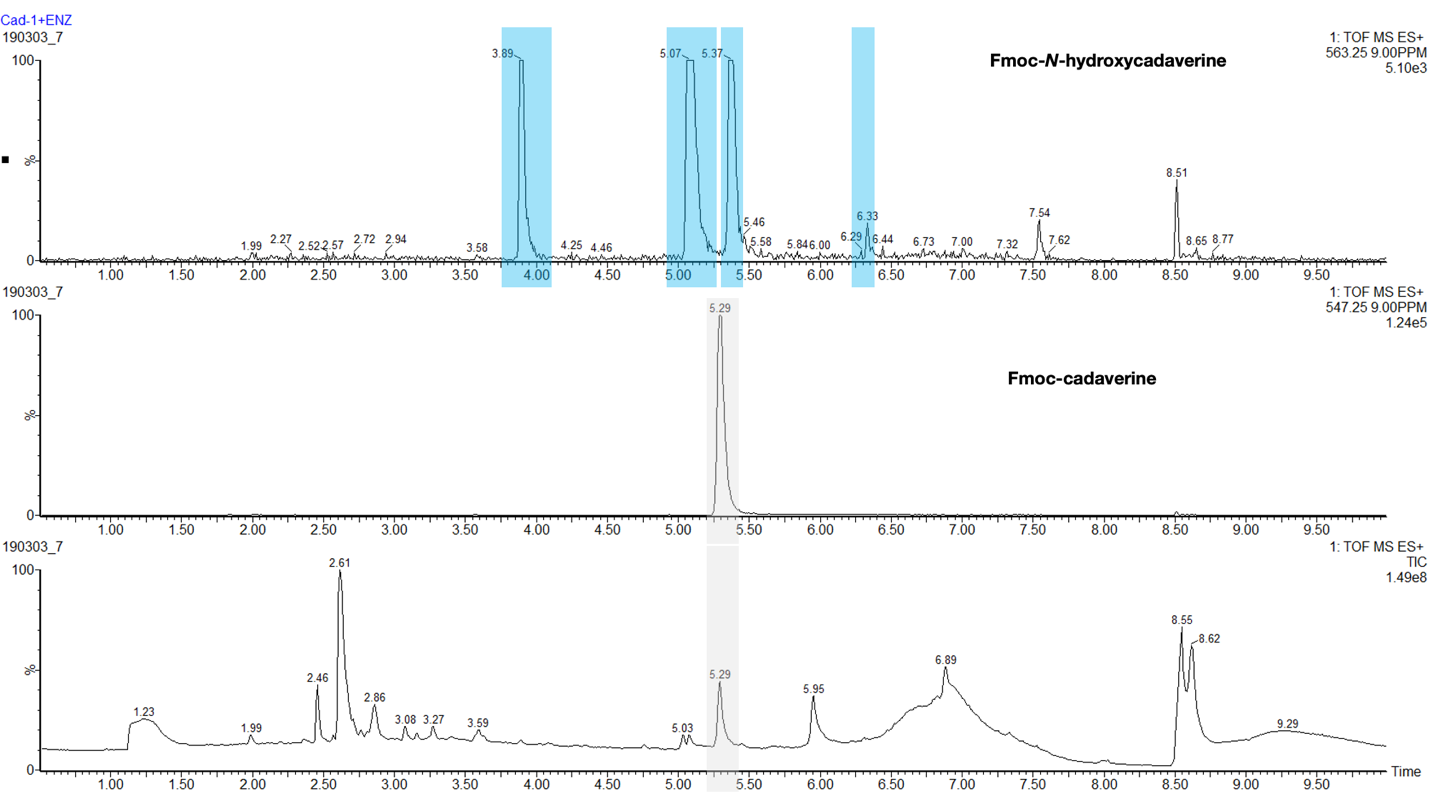
)

B)

**Figure S12. UPLC-HRMS of cadaverine and products.** A) Extracted ion chromatogram (*m/z* 547.25 and 563.25) from assays with 50 μM FAD, 0.7 mM NADPH, 10 mM cadaverine, and 1 μM *Ss*DesB. Fmoc-*N*-hydroxycadaverine stereoisomers are in blue boxes and fmoc-cadaverine is in the grey box. B) Chemical formula of the *N*-hydroxycadaverine product (*m/z* 563.2563) shown in the blue box was determined to be C_35_H_34_N_2_O_5_H within 3 ppm.
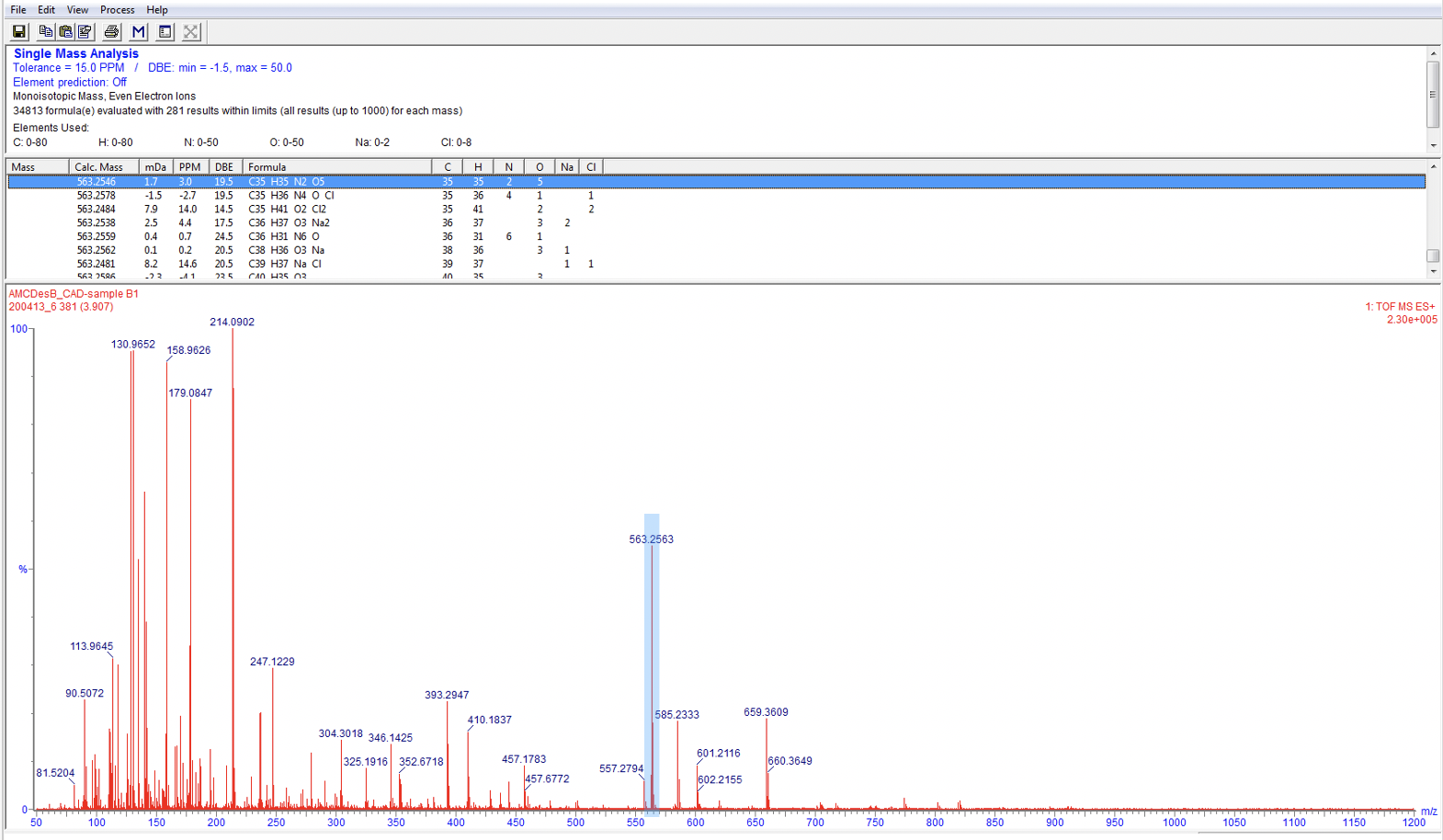


A
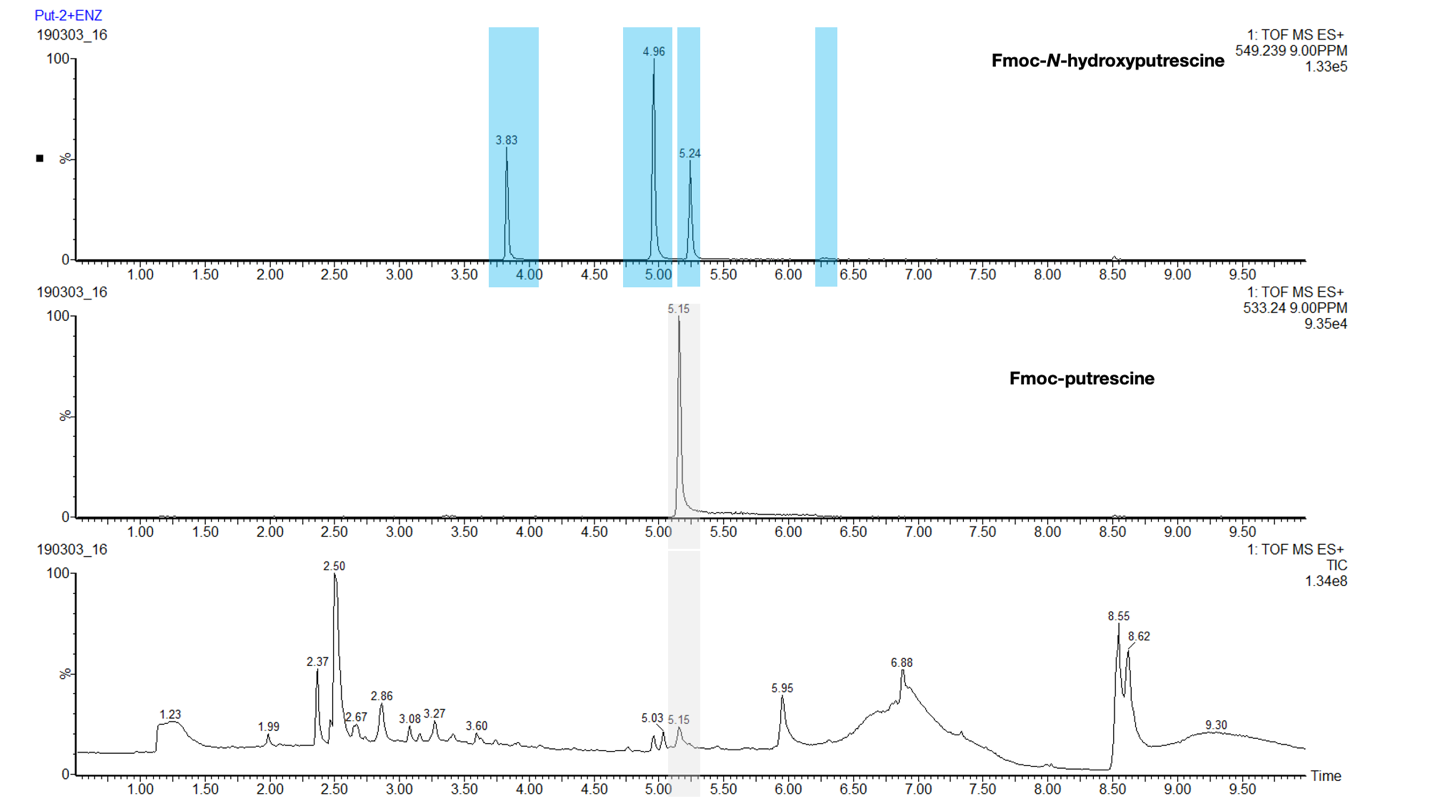
)

B)


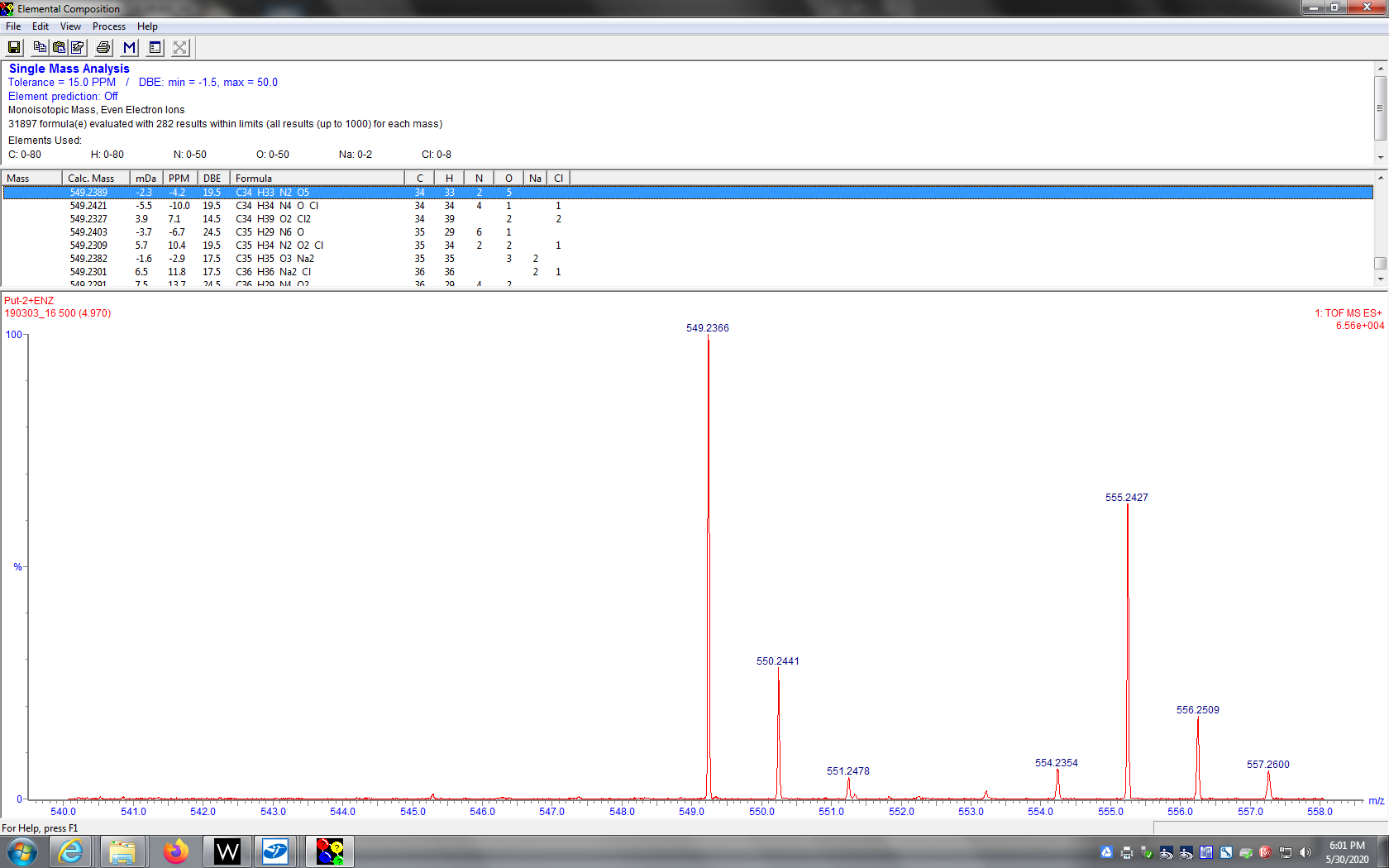


**Figure S13. LC/MS of putrescine and products.** A) Extracted ion chromatogram (*m/z* 533.24 and 549.24) from LC/MS of assays with 50 μM FAD, 0.7 mM NADPH, 10 mM putrescine, and 1 μM *Ss*DesB. Fmoc-*N*-hydroxyputrescine stereoisomers are in blue boxes and fmoc-putrescine is in the grey box. B) Chemical formula of the *N*-hydroxyputrescine product (*m/z* 549.24) shown in the blue box was determined to be C_34_H_32_N_2_O_5_H within 4.2 ppm.

A)
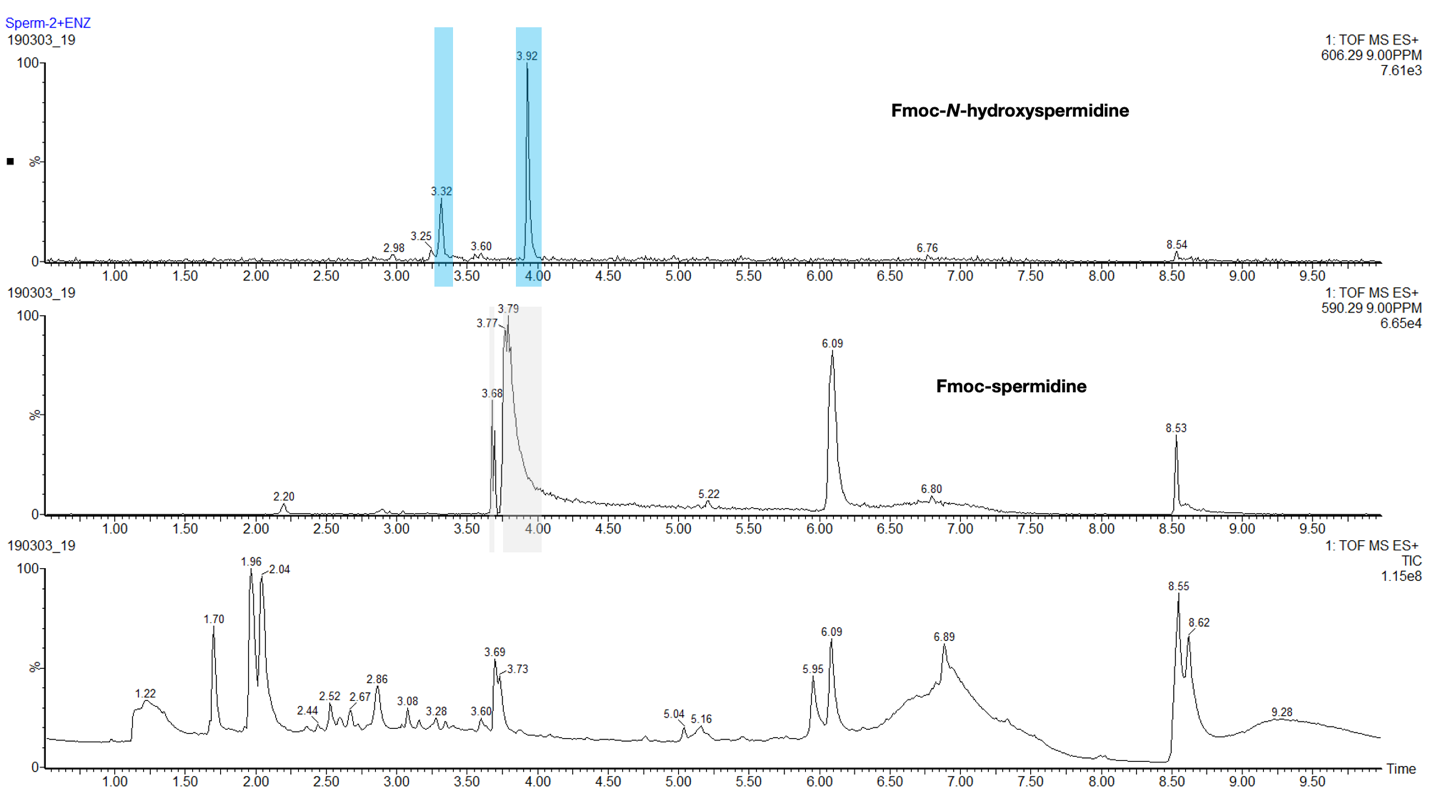


B)


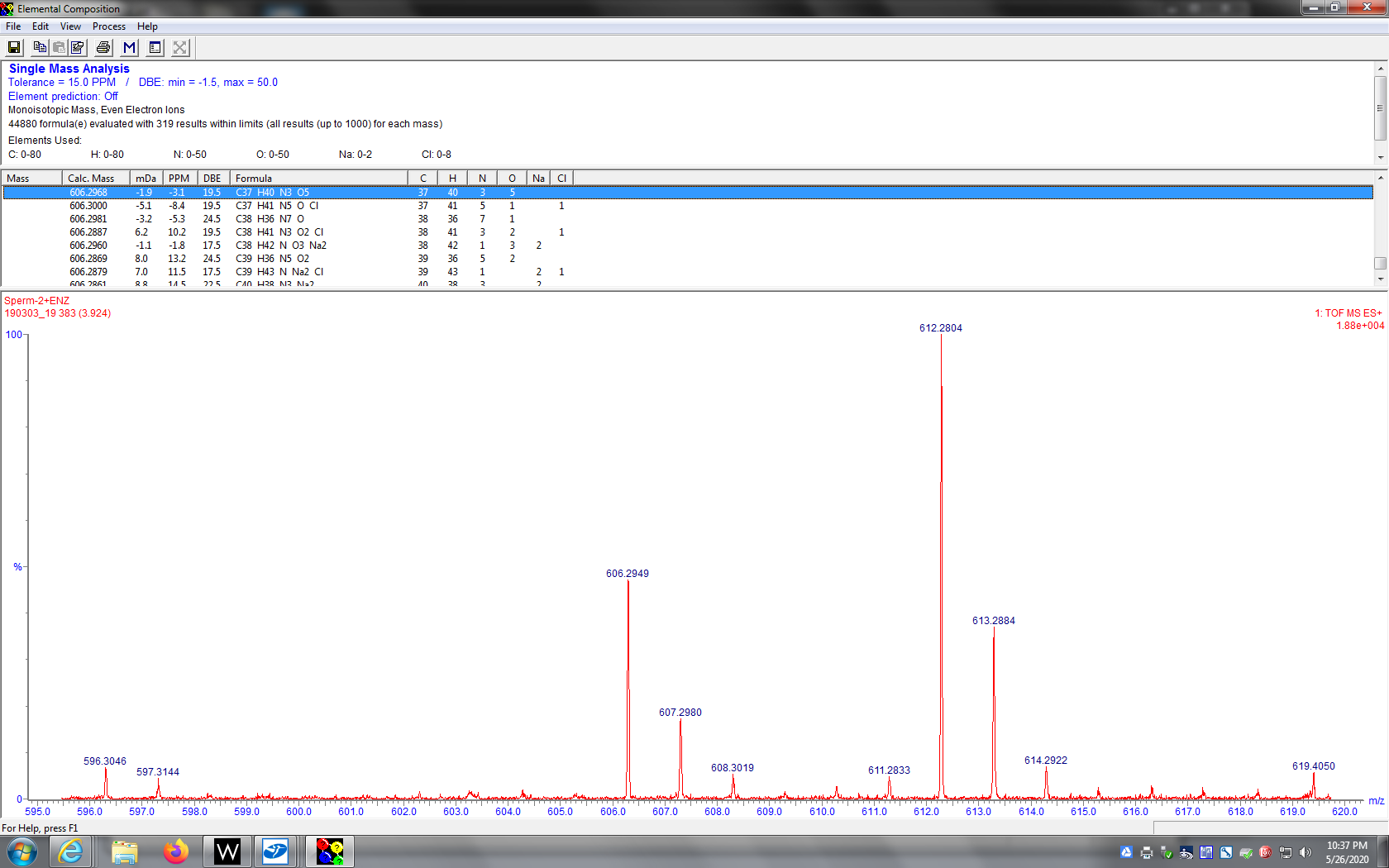


**Figure S14. UPLC-HRMS of spermidine and products.** A) Extracted ion chromatogram (*m/z* 547.25 and 563.25) from LC/MS of assays with 50 μM FAD, 0.7 mM NADPH, 10 mM spermidine, and 1 μM *Ss*DesB. Fmoc-*N*-hydroxyspermidine stereoisomers are in blue boxes and fmoc-spermidine is in the grey box. B) Chemical formula of the *N*-hydroxyspermidine product (*m/z* 606.2949) shown in the blue box was determined to be C_37_H_39_N_3_O_5_H within 3.1 ppm.

A)
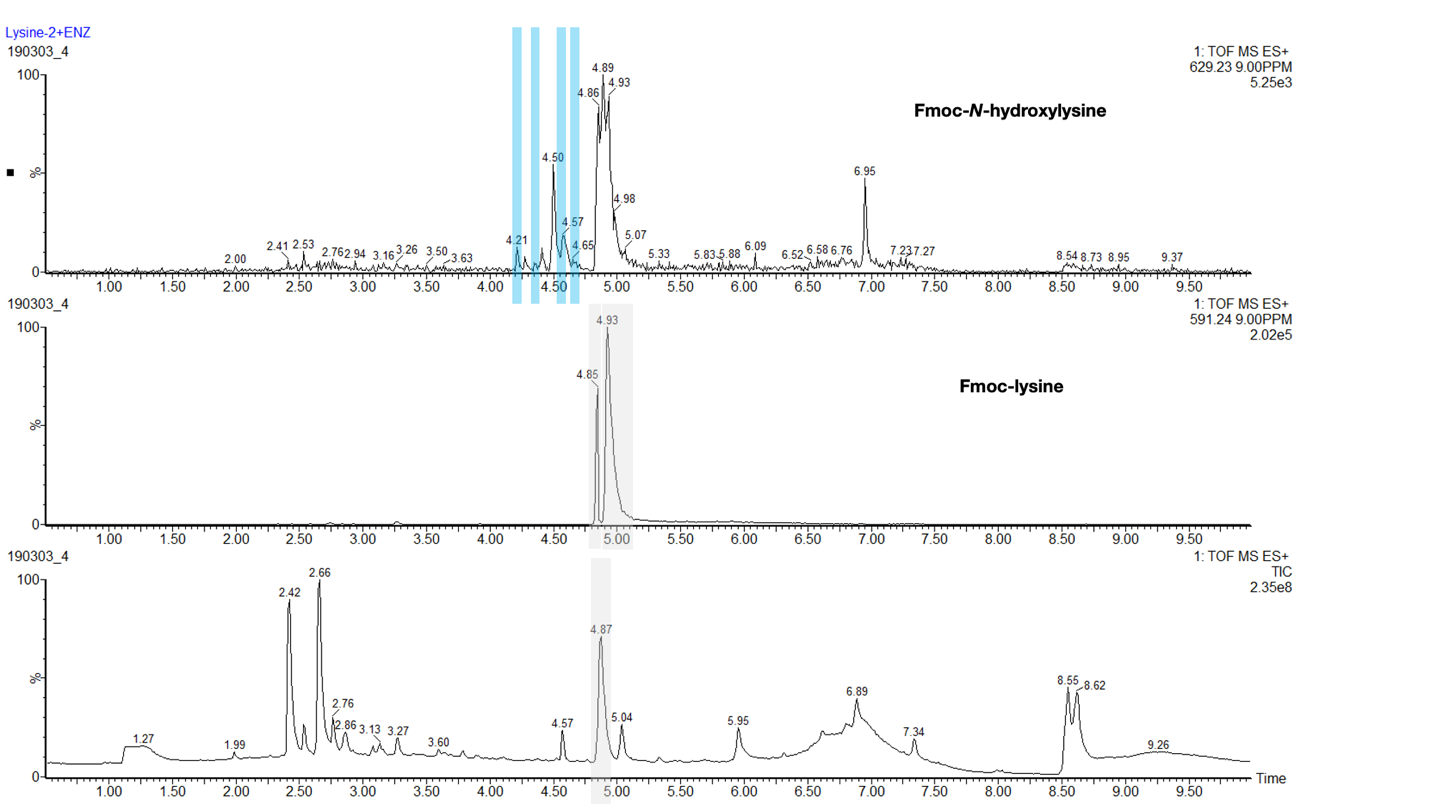


B)


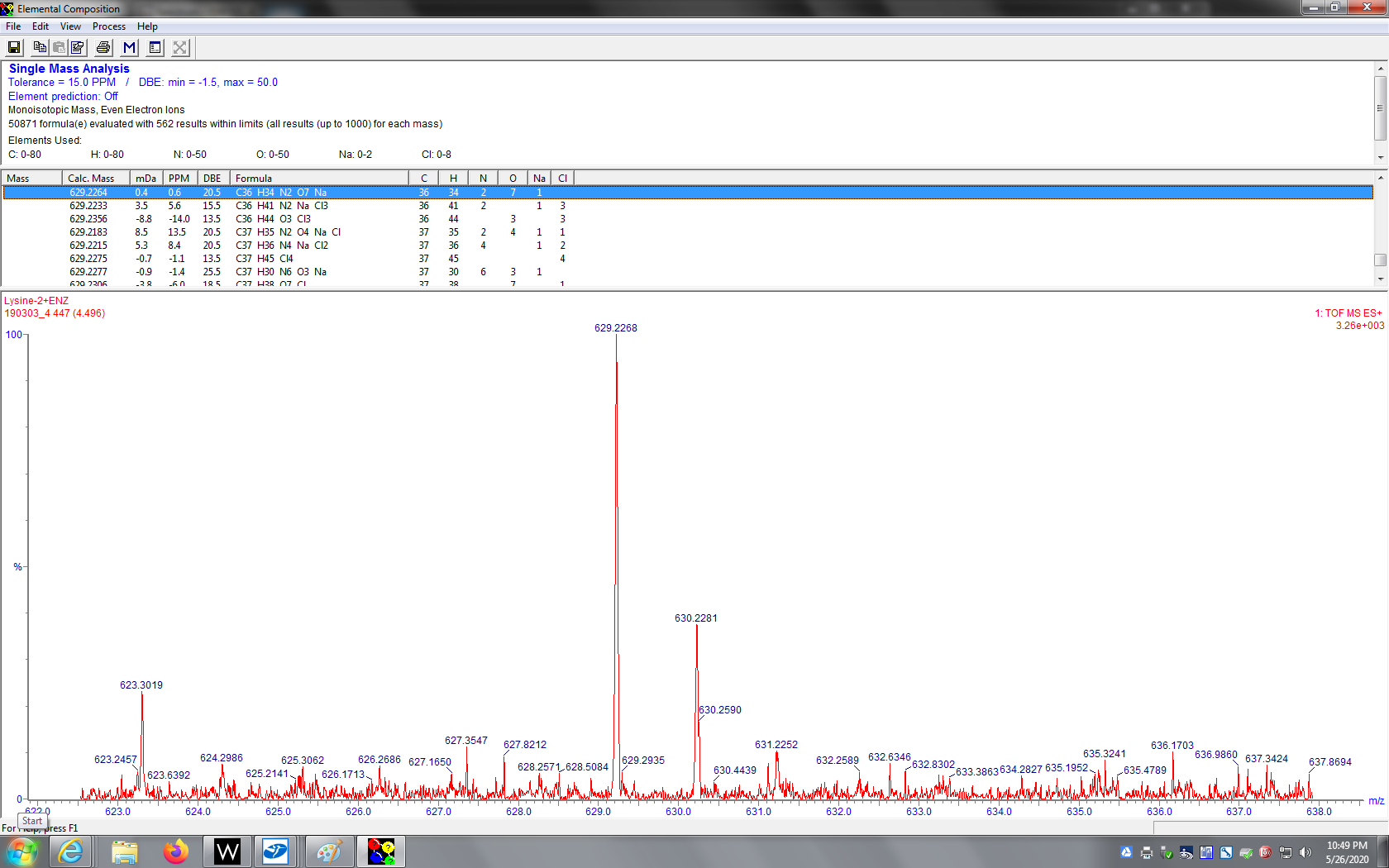


**Figure S15. UPLC-HRMS of lysine and products.**A) Extracted ion chromatogram (*m/z* 591.24 and 629.23) from LC/MS of assays with 50 μM FAD, 0.7 mM NADPH, 10 mM L-lysine, and 1 μM SsDes B. Fmoc-*N*-hydroxylysine stereoisomers are in blue boxes and fmoc-lysine is in grey box. B) Chemical formula of the *N*-hydroxylysine product (*m/z* 629.2268) shown in the blue box was determined to be C_36_H_34_N_2_O_7_Na within 0.6 ppm.


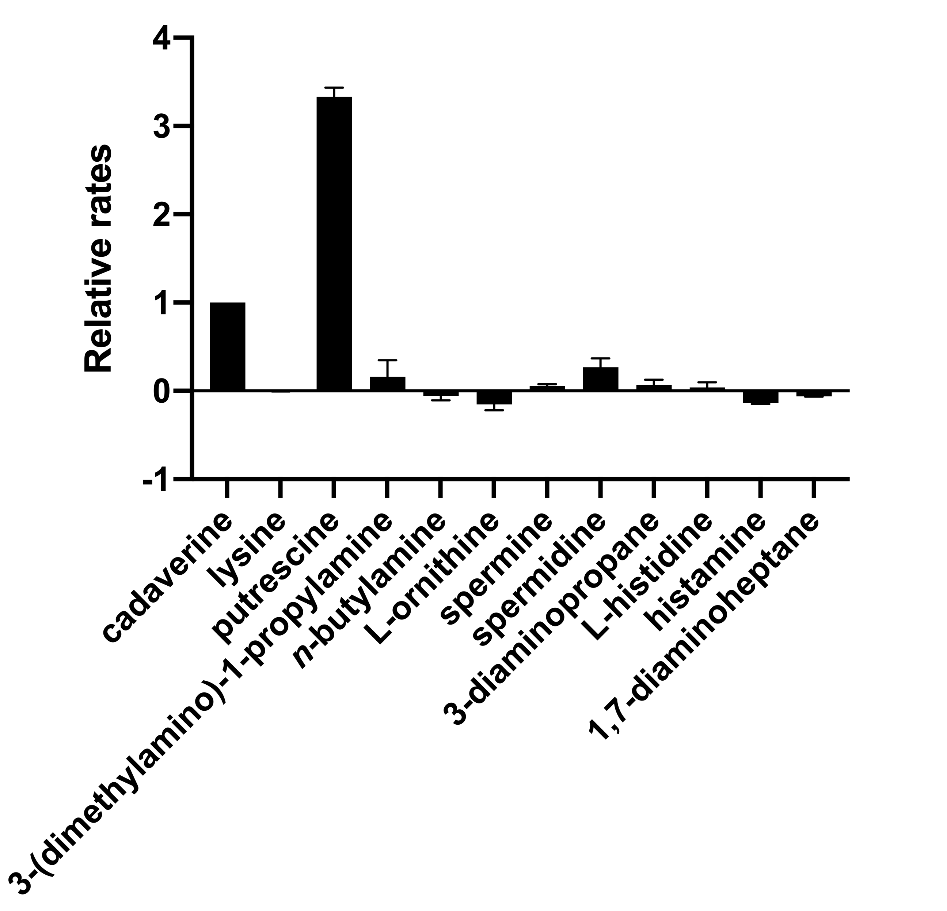


**Figure S16. Relative rates of *N*-hydroxylation determined in product formation assays with *Ss*DesB with an *N*-terminal hexahistidine tag.** Initial rates measured with 10 mM L-lysine, cadaverine, spermidine, putrescine, *n*-butylamine, L-ornithine, 1,3-diaminopropane, 1,7-diaminoheptane, histamine, spermine, 3-propylamine, and L-lysine in product formation assays in the presence of *Ss*DesB without an *N*-terminal histidine tag, 50 µM FAD, and 0.7 mM NADPH. *n*-Butylamine, L-ornithine, 1,3-diaminopropane, 1,7-diaminoheptane, histamine, spermine, 3-propylamine, and L-lysine did not yield any Fmoc-derivitized *n*-hydroxylated products by LC/MS. Assays were performed in triplicate and normalized to the initial rate of cadaverine.

| Table S1: Structure Alignments of *Ss*DesB with homologs | | | | | |
| --- | --- | --- | --- | --- | --- |
| Protein | PDB code | Z-score^*^ | r.m.s.d^&^ | N_align_^#^ | Sequence identity % |
| *E. amylovora* DfoA | 508p, chain B | 19.9 | 0.85 | 407 | 53 |
| *E. amylovora* DfoA complex with NADP^+^ | 508r, chain B | 19.7 | 0.87 | 427 | 53 |
| *Kutzneria sp.* 144 KtzI complex with NADP^+^ and ornithine | 4tm0, chain C | 15.7 | 1.77 | 361 | 30 |
| *Kutzneria sp.* 144 KtzI complex with NADP^+^ and ornithine and Bromide ion | 4tm1, chain B | 15.1 | 1.74 | 364 | 29 |
| *Pseudomonas aeruginosa* ornithine hydroxylase (PvdA) | 3s5w, chain A | 13.2 | 1.96 | 361 | 29 |
| *Aspergillus fumigatus* ornithine hydroxylase SidA N263A complex with NADP^+^ and ornithine | 5cku, chain A | 12.9 | 2.08 | 362 | 30 |
| Structural alignments were performed using PDBeFold ([www.ebl.ac.uk/msd-srv/ssm/](http://www.ebl.ac.uk/msd-srv/ssm/)) using chain A of *Ss*DesB in complex with NADP^+^ (PDB code: 6XBB).  ^*^The *Z* score measures the statistical significance of a match in terms of Gaussian statistics. The higher the *Z* score, the higher the statistical significance of the match  ^&^Root-mean-square deviation  ^#^Number of matched residues | | | | | |

**References**

[1] Kumar S, Stecher G, Tamura K. MEGA7: molecular evolutionary genetics analysis version 7.0

for bigger datasets. Mol Biol Evol. 2016;33(7):1870-4.

[2] Madeira F, Park YM, Lee J, Buso N, Gur T, Madhusoodanan N, et al. The EMBL-EBI search and

sequence analysis tools APIs in 2019. Nucleic Acids Res. 2019;47(W1):W636-W41.
