# Supplementary material for: Characterization of a broadly specific cadaverine N-hydroxylase involved in desferrioxamine B biosynthesis in Streptomyces sviceus
Source: PLoS One. 2021 Mar 30;16(3):e0248385. doi: 10.1371/journal.pone.0248385 (PMC8009421; doi:10.1371/journal.pone.0248385)
Supplement: S1 Raw images — (PDF) [file pone.0248385.s002.pdf]

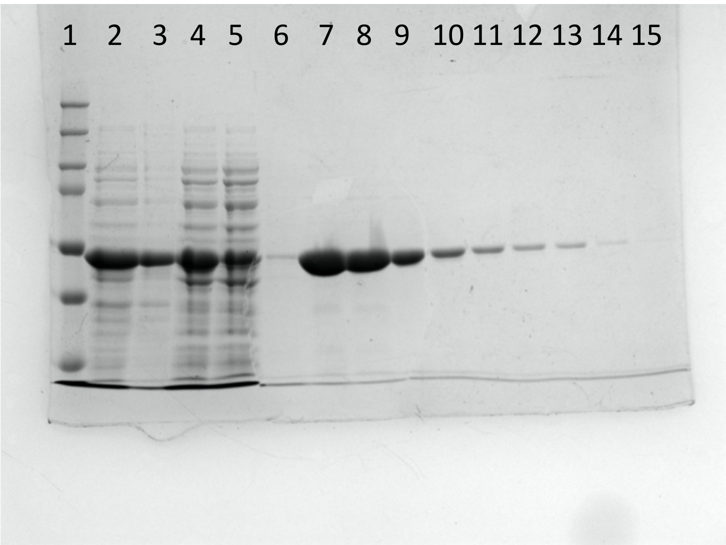

Original gel for Figure S4. This Coomassie-stained SDS-PAGE gel of protein fractions collected during the purification of *Ss*DesB was taken using the Coomassie Fluor Orange method using no filter on a Bio-Rad ChemiDoc imaging system with Universal Hood II. The lanes represent the following: lane 1, molecular mass marker; lane 2, crude lysate; lane 3, pellet; lane 4, supernatant; lane 5, flow-through; lane 6, wash; and lanes 7–15, elution. Lanes 1–7 were shown in Fig S4 below.

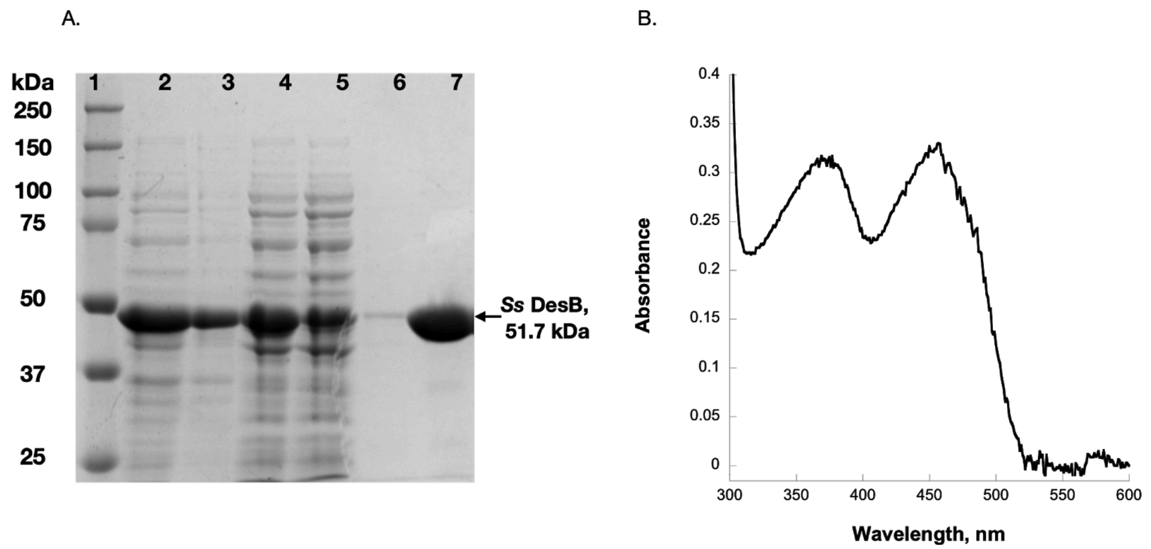

Final Gel Figure S4. A) SDS-PAGE of *Ss*DesB purification. Lane 1, molecular mass marker; lane 2, crude lysate; lane 3, pellet; lane 4, supernatant; lane 5, flow-through; lane 6, wash; lane 7, elution. B) UV-visible spectrum of FAD bound to purified *Ss*DesB.
